# The combination of procalcitonin and C-reactive protein or presepsin alone improves the accuracy of diagnosis of neonatal sepsis: a meta-analysis and systematic review

**DOI:** 10.1186/s13054-018-2236-1

**Published:** 2018-11-21

**Authors:** Lin Ruan, Guan-Yu Chen, Zhen Liu, Yun Zhao, Guang-Yu Xu, Shu-Fang Li, Chun-Ni Li, Lin-Shan Chen, Zheng Tao

**Affiliations:** 1 Departments of Anesthesiology, Guangxi Medical University Affiliated Tumor Hospital, Naning, Guangxi China; 2 Departments of Respiratory Oncology, Guangxi Medical University Affiliated Tumor Hospital, Naning, Guangxi China; 3 Departments of Urology, Guangxi Medical University Affiliated Tumor Hospital, Naning, Guangxi China

**Keywords:** Meta-analysis, Systematic review, Procalcitonin, C-reactive protein, Neonatal sepsis

## Abstract

**Background:**

Sepsis is an important cause of neonatal morbidity and mortality; therefore, the early diagnosis of neonatal sepsis is essential.

**Method:**

Our aim was to compare the diagnostic accuracy of procalcitonin (PCT), C-reactive protein (CRP), procalcitonin combined with C-reactive protein (PCT + CRP) and presepsin in the diagnosis of neonatal sepsis. We searched seven databases to identify studies that met the inclusion criteria. Two independent reviewers performed data extraction. The pooled sensitivity, specificity, positive likelihood ratio (PLR), negative likelihood ratio (NLR), diagnostic odds ratio (DOR), area under curve (AUC), and corresponding 95% credible interval (95% CI) were calculated by true positive (TP), false positive (FP), false negative (FN), and true negative (TN) classification using a bivariate regression model in STATA 14.0 software. The pooled sensitivity, specificity, PLR, NLR, DOR, AUC, and corresponding 95% CI were the primary outcomes. Secondary outcomes included the sensitivity and specificity in multiple subgroup analyses.

**Results:**

A total of 28 studies enrolling 2661 patients were included in our meta-analysis. The pooled sensitivity of CRP (0.71 (0.63, 0.78)) was weaker than that of PCT (0.85 (0.79, 0.89)), PCT + CRP (0.91 (0.84, 0.95)) and presepsin (0.94 (0.80, 0.99)) and the pooled NLR of presepsin (0.06 (0.02, 0.23)) and PCT + CRP (0.10 (0.05, 0.19)) were less than CRP (0.33 (0.26, 0.42)), and the AUC for presepsin (0.99 (0.98, 1.00)) was greater than PCT + CRP (0.96 (0.93, 0.97)), CRP (0.85 (0.82, 0.88)) and PCT (0.91 (0.89, 0.94)). The results of the subgroup analysis showed that 0.5–2 ng/mL may be the appropriate cutoff interval for PCT. A cut-off value > 10 mg/L for CRP had high sensitivity and specificity.

**Conclusions:**

The combination of PCT and CRP or presepsin alone improves the accuracy of diagnosis of neonatal sepsis. However, further studies are required to confirm these findings.

**Electronic supplementary material:**

The online version of this article (10.1186/s13054-018-2236-1) contains supplementary material, which is available to authorized users.

## Introduction

Sepsis is a major cause of neonatal morbidity and mortality [1, 2, 3]. In routine clinical practice, the rapid and accurate diagnosis of neonatal sepsis is often difficult because the clinical presentation of neonatal sepsis may be confused with non-infectious disorders, the onset of sepsis may be acute, and the clinical process can quickly subside. Improving the accuracy of diagnostic testing may improve outcomes in those with true sepsis and decrease the indiscriminate use of antibiotics in those without sepsis [4]. Microbial cultures can help identify serious bacterial infections, but these often produce false negative results, especially after maternal use of antibiotics and may produce false positive results due to sample contamination. In addition, microbial cultures have a time delay (2–3 days) in obtaining results. Therefore, neonates with clinical manifestations of sepsis or risk factors for serious bacterial infections are usually treated with antibiotics while waiting for the results of microbiology testing [5]. This inevitably leads to the overuse of antibiotics, which in turn may lead to the emergence of multiple drug-resistant bacteria in the neonatal intensive care unit (NICU) [3, 6]. Therefore, to prevent microbial resistance due to unnecessary empirical treatment and to avoid unnecessary hospitalization, a definitive diagnosis should be ensured based on laboratory tests with higher diagnostic value [7]. Biomarkers can be important in the timely diagnosis of sepsis, helping in the differential diagnosis of non-infectious diseases and decision-making in initial treatment. C-reactive protein (CRP) is produced by the liver in response to inflammation and/or infectious stimuli, and thus it is considered to be an acute-phase protein [8, 9]. CRP may also be increased in some antenatal conditions, such as fetal distress, stress delivery, and maternal fever, in the absence of systemic infection [8]. Therefore, its specificity is low, and it is preferably used in combination with another serum biomarker. Procalcitonin (PCT) appears to be one of the most promising among the different molecules studied as biomarkers of sepsis. PCT is a procalcitonin precursor protein produced by monocytes and hepatocytes. After exposure to bacterial endotoxin, PCT levels within 2–4 h rise sharply, within 6–8 h they reach a plateau, and then they return to normal levels after 24 h [8, 10]. Serum PCT levels appear to correlate with the severity of the microbial attack and rapidly decrease after appropriate antibiotic treatment. In contrast to CRP, local bacterial infections, severe viral infections, and inflammatory reactions of non-infectious origin are either not associated with increased PCT or are only associated with a slight increase in PCT. In healthy and preterm neonates, there is a physiological increase in serum PCT after birth that peaks at 24 h of age [8, 10]. Presepsin is a nicked truncated form of soluble CD14 (sCD14), which is released by detachment from the surface of immune cells after stimulation by pathogens. Recently, presepsin has been described as a reliable diagnostic and prognostic marker for neonatal sepsis. Based on the above considerations, we performed a meta-analysis to compare the diagnostic accuracy of PCT, CRP, PCT combined with CRP, and presepsin in diagnosing neonatal sepsis.

## Methods

This study was performed according to the Preferred Reporting Items for Systematic Reviews and Meta-Analyses (PRISMA) statement. The protocol for this meta-analysis is available in PROSPERO (CRD 42018091339).

### Search for trials

We searched PubMed, Web of Science, Cochrane Library, Embase, China National Knowledge Infrastructure (CNKI), Wanfang, and Weipu databases from their inception dates to 16 Aug. 2018 using the keywords “procalcitonin,” “C-reactive protein,” and “presepsin” to identify studies that met the inclusion criteria. There were no restrictions on language. The detailed search strategy is presented in Additional file 1: Table S1.

### Selection criteria

Studies were selected based on the following inclusion criteria: (1) neonatal patients with sepsis as the experimental group, whereas the participants with non-sepsis (the patient is suspected of having sepsis but has no sepsis) were regarded as the control group; (2) enough data to calculate the outcome data (true positive (TP), false positive (FP), true negative (TN), false negative (FN)); (3) the participants were diagnosed using the gold standard; (4) the gold standard for diagnosis of sepsis was defined in the study; and (5) blood measurement (of PCT, CRP, PCT + CRP, or presepsin) had to be performed at the time of clinical presentation with suspected sepsis before administration of antimicrobial therapy or in asymptomatic neonates at the time of inclusion in the study. The exclusion criteria were as follows: (1) the diagnostic method for sepsis was not serum PCT, CRP, PCT + CRP, or presepsin; (2) insufficient data to calculate the outcome data (TP, FP, TN, FN); (3) sepsis was diagnosed without a gold standard; (4) studies that used measurements that were made only on maternal or umbilical cord blood samples; (5) neonates treated with antibiotics within the first 72 h; (6) studies involving healthy neonates as controls; and (7) abstracts, reviews, and animal experiments.

### Data extraction

Two researchers independently extracted the following information from each study: name of study, year; design country; region; assay method; test time; cutoff; study period; age (days); gestational age (weeks); weight (g); sepsis onset; characteristics and number of patients; and outcome data (TP, FP, FN, and TN). Discrepancies were resolved by consensus.

### Risk-of-bias assessments

The analysis of risk of bias and applicability of diagnostic accuracy for the studies included was assessed independently by the two researchers based on the Quality Assessment of Diagnostic Accuracy Studies (QUADAS-2) by RevMan (version 5.2, Cochrane Collaboration, Oxford, UK). QUADAS-2 consists of four sections: patient selection, index test, reference standard, and flow and timing. The studies included were graded as low risk, high risk, or unclear bias based on the following criteria: (1) if the answers to all of the questions for a section were “yes,” then the risk of bias was judged as “low;” (2) if any answer to a question in a section was “no,” then risk of bias was judged as “high;” (3) the unclear bias was only to be used when insufficient information was provided. Applicability was judged as low, high, or unclear with the above criteria.

### Statistical analysis

Threshold effects were calculated by testing Spearman correlation using Meta-DiSc (version 1.4) software, and *P* values <0.05 represent significant threshold effects. *I*^2^ and a bivariate boxplot were used to measure the heterogeneity caused by non-threshold effects. If the *I*^2^ value was ≥ 50% and the *P* value ≤0.05, or there were studies that fell outside the bivariate boxplot, indicating that the heterogeneity was significant due to the non-threshold effect, then meta-regression analysis to find sources of heterogeneity was performed. The pooled sensitivity, specificity, positive likelihood ratio (PLR), negative likelihood ratio (NLR), diagnostic odds ratio (DOR), AUC, and corresponding 95% credible interval (CI) were calculated by TP, FP, FN, and TN using a bivariate regression model using STATA 14.0 software. Deek’s funnel plot was used to detect publication bias, with *P* < 0.05 indicating publication bias. The visual presentation of diagnostic performance was assessed by the Fagan plot. We performed two methods to evaluate if there was a significant difference in sensitivity, specificity, PLR, NLR, or AUC between any two diagnostic biomarkers. The qualitative method is to observe whether the 95% confidence intervals between different statistical indicators overlap. If there is overlap, there is no statistical significance. Quantitative tests are based on the *z*-test:

$$ \left({\mathrm{X}}_1-{\mathrm{X}}_2\right)/{\left({{\mathrm{SE}}_1}^2+{{\mathrm{SE}\mathrm{X}}_2}^2\right)}^{1/2}, $$

where X_1_ and X_2_ represent the AUC, and SE_1_ and SE_2_ are the corresponding standard errors, respectively. If the *P* value obtained from the *z*-test is less than *p*’ (*p*’ = 0.05/6), then it is considered there is a statistically significant difference between statistical indicators.

## Results

### Studies retrieved and their characteristics

**Fig. 1:**
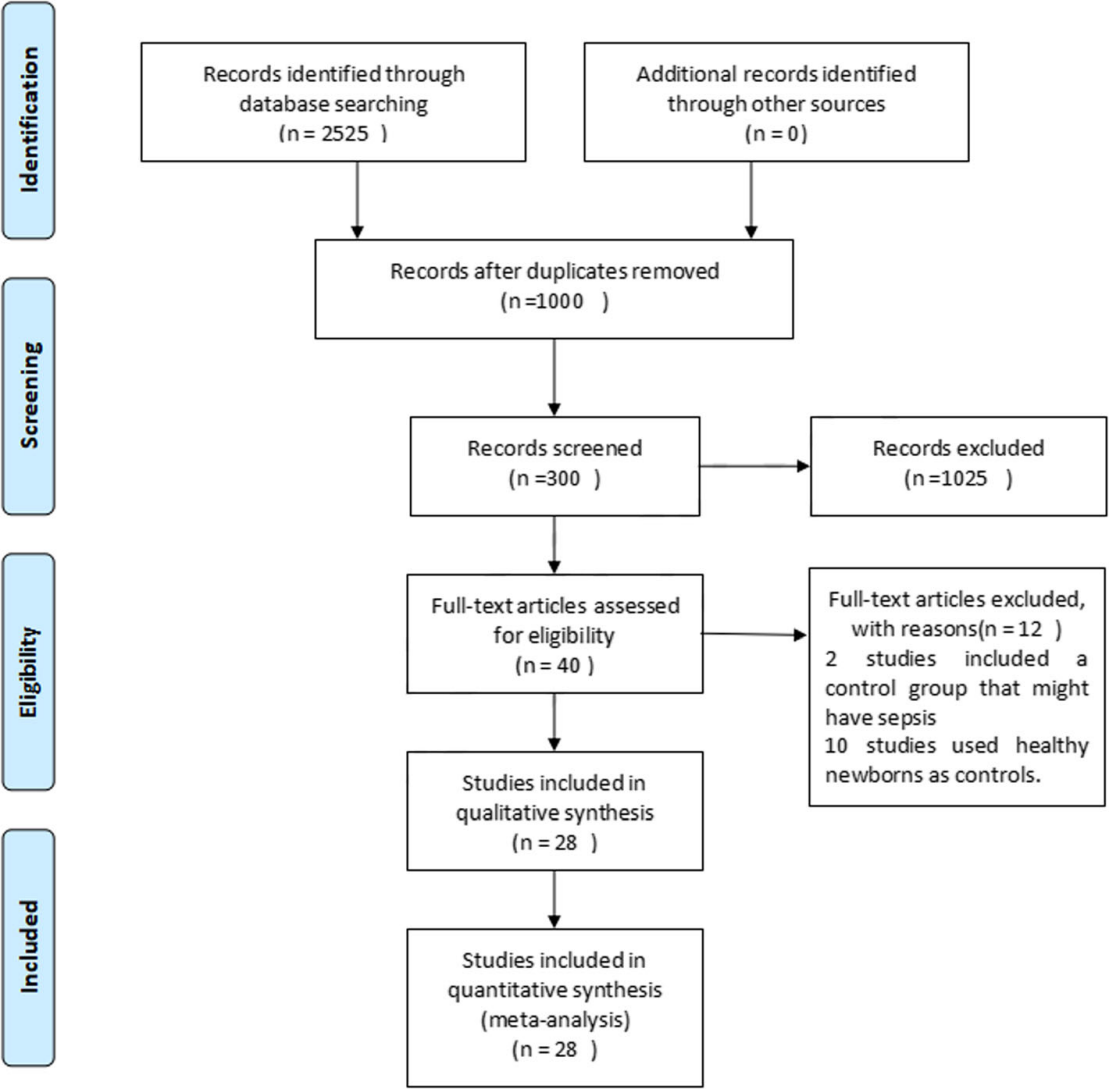
Literature search and screening process

The database search identified 2525 records that potentially qualified for inclusion. The titles and abstracts of these records were then filtered. Full texts of 300 records were screened, and 40 met the inclusion criteria. Additional file 1: Table S2 lists the main characteristics of the 40 studies included in the meta-analysis. Of the 40 studies included, two studies included a control group that might have sepsis, and ten studies used healthy neonates as controls. We did not include these studies in the meta-analysis based on the inclusion criteria provided. Eventually, 28 studies (2661 participants) were included in the meta-analysis (Fig. 1), of which 9 studies were not written in English (Additional file 1: Table S1).

Overall, 1281 participants were assigned to the sepsis group and 1380 to the non-sepsis group. In terms of region, 17 (60.7%) trials recruited patients from Asia, 8 (28.6%) from Europe, 1 (3.6%) from North America, and 2 (7.1%) from Africa.

In terms of sepsis onset, three studies included only patients with early-onset neonatal sepsis (diagnosed in the first 72 h of life), five studies included patients with late-onset neonatal sepsis (diagnosed after 72 h of life), and the remaining trials included early-onset and late-onset neonatal sepsis or did not provide relevant information.

In terms of trial design, 13 studies were prospective cohort studies, 12 studies were case-control studies, and 3 studies were cross-sectional studies.

### Risk-of-bias assessments

**Fig. 2:**
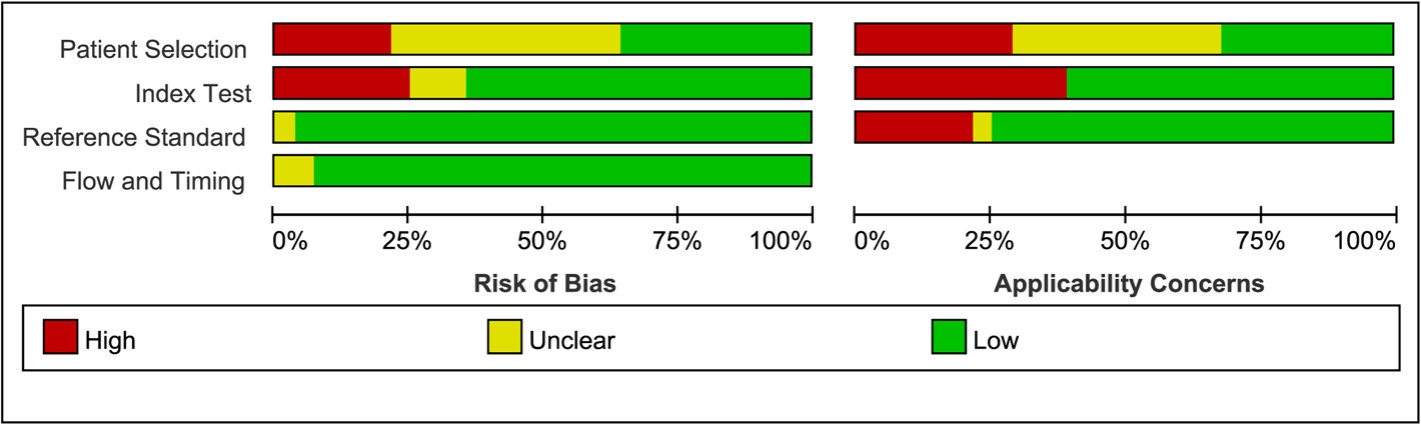
Risk of bias and applicability concerns

**Fig. 3:**
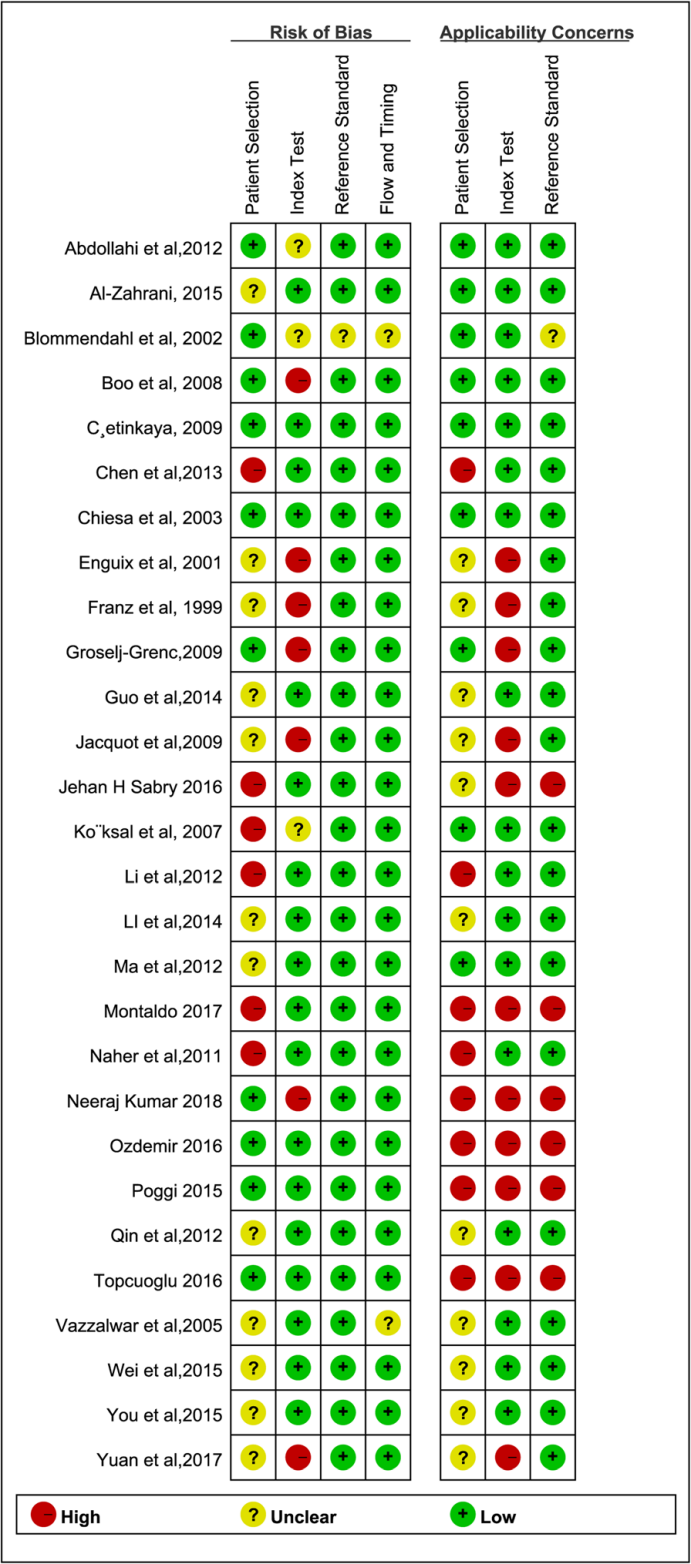
Risk of bias and applicability concerns - summary

Figures 2 and 3 show the results of assessment for risk of bias. In terms of the risk of bias, of the 28 studies included in our meta-analysis, 12 studies had unclear bias in patient selection. There were 18 studies that were judged as having low bias in the index tests, 27 studies were allocated as having low bias in terms of reference standards, and 26 studies were judged as having low bias in terms of flow and timing. In terms of applicability concerns, 8 studies had high bias in patient selection, 17 studies were judged as having low bias in relation to index tests, and 21 studies were classified as causing high concern about reference standards.

### Threshold effect and heterogeneity

The Spearman correlation coefficient and *P* value for PCT, CRP, PCT + CRP, and presepsin were 0.144 and 0.523, 0.301 and 0.174, 0.433 and 0.244, and 0.371 and 0.468, respectively, which indicated that there was no significant threshold effect, and thus we combined the sensitivity, specificity, PLR, NLR, DOR, and AUC. We used *I*^2^ and a bivariate boxplot (Additional file 2: Figure S1) to measure the heterogeneity caused by non-threshold effects. For PCT, CRP, PCT + CRP and presepsin, the *I*^2^ values were 96%, 98%, 0%, and 80%, respectively.

### Forest plot and area under the summary ROC (SROC) curve

**Fig. 4:**
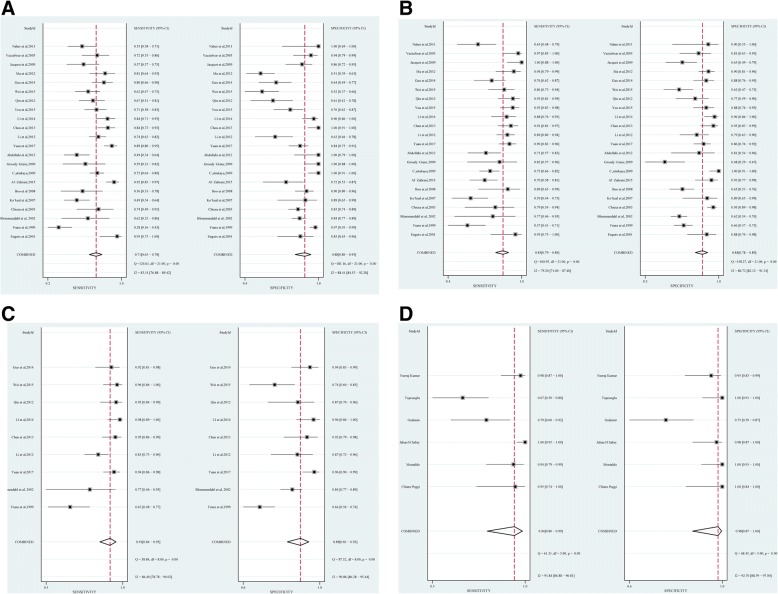
Sensitivity and specificity. **a** C-reactive protein (CRP). **b** Procalcitonin (PCT). **c** PCT plus CRP. **d** Presepsin. Point estimates for sensitivity and 95% confidence intervals are shown with pooled estimates. Q = Cochran Q statistic

**Fig. 5:**
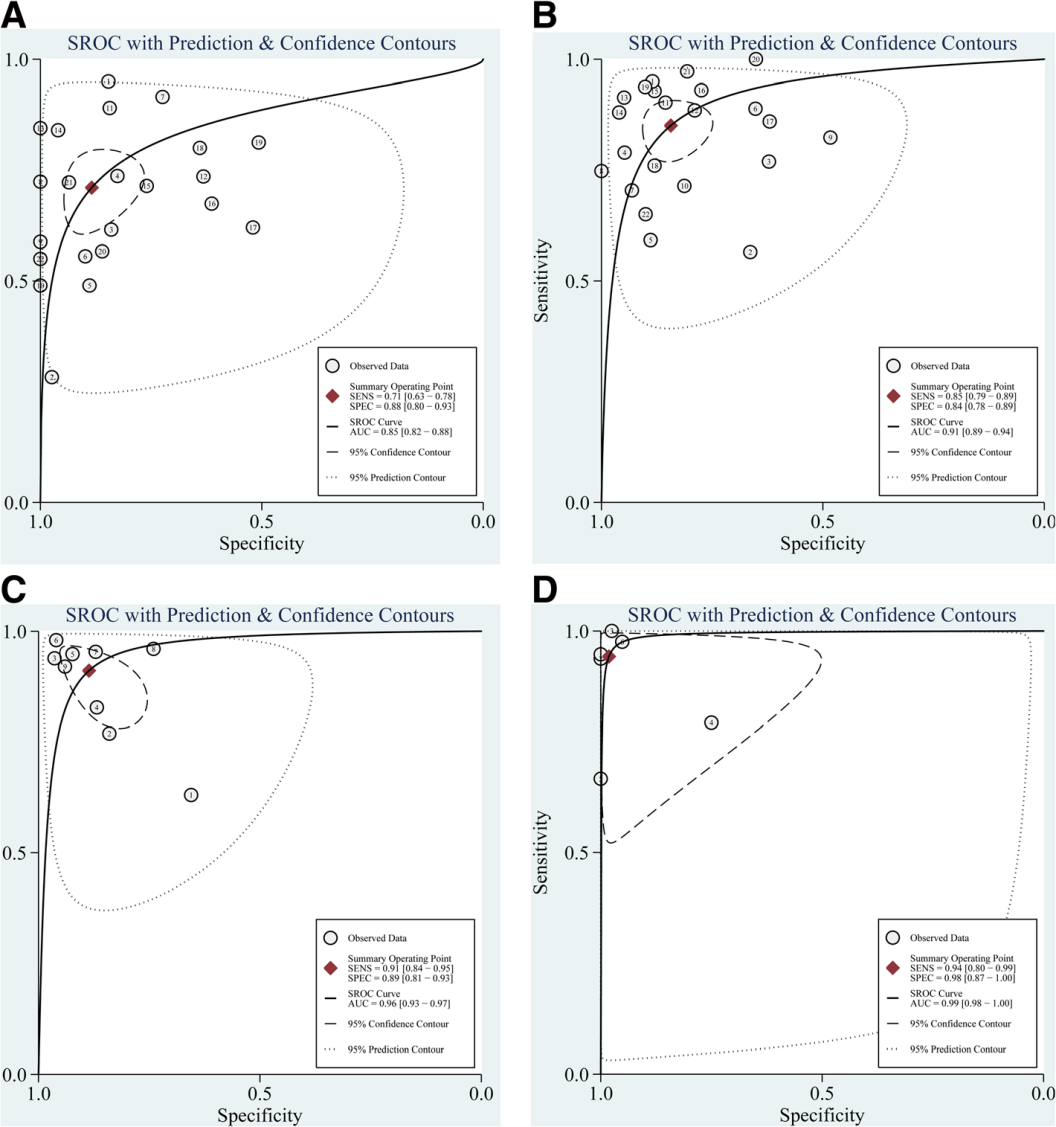
Summary receiver-operating characteristic (SROC) curves for the diagnosis of neonatal sepsis. **a** C-reactive protein (CRP). **b** Procalcitonin (PCT). **c** PCT plus CRP. **d** Presepsin. AUC = area under the curve

Forest plots of sensitivity and specificity are shown in Fig. 4. Additional file 1: Table S3 shows the pooled results of PCT, CRP, PCT + CRP and presepsin. Figure 5 shows the SROC curve for the diagnosis of neonatal sepsis. The pooled sensitivity, specificity, PLR, NLR, DOR, AUC and corresponding 95% CI (95% CI) of PCT, CRP, PCT + CRP, and presepsin were 0.85 (0.79, 0.89), 0.71 (0.63, 0.78), 0.91 (0.84, 0.95), 0.94 (0.80, 0.99); 0.84 (0.78, 0.89), 0.88 (0.80, 0.93), 0.89 (0.81, 0.93), 0.98 (0.87, 1.00); 5.4 (3.7, 7.9), 6.1 (3.6, 10.5), 8.0 (4.6, 14.0), 50.8 (6.5, 394.7); 0.18 (0.13, 0.25), 0.33 (0.26, 0.42), 0.10(0.05, 0.19), 0.06 (0.02, 0.23); 31 (17, 54), 19 (10, 35), 79 (26, 246), 864 (65, 11473); and 0.91(0.89–0.94), 0.85 (0.82–0.88), 0.96 (0.93–0.97), 0.99 (0.98–1.00), respectively.

### Pair-wise comparisons

Additional file 1: Table S3 shows the results of pair-wise comparisons between statistical indicators for sensitivity, specificity, PLR, NLR, and AUC. The pooled sensitivity of CRP (0.71 (0.63, 0.78)) was weaker than that of PCT (0.85 (0.79, 0.89)), PCT + CRP (0.91 (0.84, 0.95)) and presepsin (0.94 (0.80, 0.99)) and the pooled NLR of presepsin (0.06 (0.02, 0.23)) and PCT + CRP (0.10 (0.05, 0.19)) were less than for CRP (0.33 (0.26, 0.42)), and the AUC of presepsin (0.99 (0.98–1.00)) was greater than for PCT + CRP (0.96 (0.93–0.97)), CRP (0.85 (0.82–0.88)), and PCT (0.91 (0.89–0.94)).

### Likelihood ratio scattergram

For PCT and CRP, the summary LRP and LRN for index testing were on the right lower quadrant (RLQ), indicating that PCT or CRP could not exclude or confirm neonatal sepsis (Additional file 3: Figure S2). For PCT + CRP, the summary LRP and LRN for index testing was between the left lower quadrant (LLQ) and right lower quadrant (RLQ), suggesting that PCT + CRP may exclude but not confirm neonatal sepsis (Additional file 3: Figure S2). For presepsin, the summary LRP and LRN for index testing was between the left upper quadrant (LUQ), indicating that presepsin could exclude and confirm neonatal sepsis (Additional file 3: Figure S2).

### Fagan diagram and publication bias

Additional file 4: Figure S3 shows the assessment of publication bias. Based on the *P* values of PCT, CRP, PCT + CRP and presepsin (0.430, 0.735, 0.825, and 0.410, respectively) and the corresponding Deek’s funnel plot, no significant publication bias was observed. Additional file 5: Figure S4 shows the Fagan diagrams. Based on the same pre-test probability of 20%, the post-test probability for presepsin (93%) was higher than for PCT + CRP (67%), PCT (58%), and CRP (60%).

### Sensitivity analysis

Sensitivity analysis was performed with a method of reducing one article at a time, and the effect of a single study on the meta-analysis was evaluated. Additional file 1: Table S10 shows the combined DOR and 95% CI calculated after deleting a single study. We observed that regardless of the excluded study, the combined DOR after removal did not significantly change, suggesting that the results of this analysis were not excessively dependent on a certain study, and our findings were robust.

### Meta-regression analysis

Meta-regression analysis of sensitivity, specificity, and joint models was performed to find potential sources of heterogeneity (Additional file 1: Tables S4, S5, and S6). According to the results of meta-regression analysis, we specified subgroups based on design, region, method, test time, and cutoff value.

### Subgroup analysis

The results of the subgroup analysis are shown in Additional file 1: Tables S7, S8, and S9.

In terms of region, PCT had similar sensitivity in Asia and Europe, while its sensitivity in North America (0.98 (0.92–1.00)) was significantly higher than in Asia (0.85 (0.80–0.91)). In Asia, the sensitivity of PCT obtained at a cutoff level of 1.53 ng/mL (0.91 (0.77–1.00)) was higher than its sensitivity at a cutoff level of 1 ng/mL (0.59 (0.26–0.91)).

For CRP, in terms of region, the sensitivity of CRP in Africa (0.92 (0.80–1.00)) was significantly higher than in Asia (0.72 (0.63–0.80)) and Europe (0.63 (0.47–0.79)). In Europe, immunonephelometric assay (0.71 (0.54–0.83)) was significantly more sensitive than chemiluminescent immunoassay (0.28 (0.01–0.54)), but its specificity (0.85 (0.81–0.89)) was significantly lower than that of chemiluminescent immunoassay (0.98 (0.92–1.00)).

For PCT + CRP, in terms of region, the sensitivity of PCT + CRP in Asia (0.93 (0.90–0.97)) was significantly higher than in Europe (0.69 (0.51–0.87)).

For presepsin, in terms of region, the specificity of presepsin in Europe (1.00 (0.94–1.00)) was significantly higher than in Asia (0.90 (0.93–0.95)). In terms of study design, the sensitivity of case-control studies (0.92 (0.80–1.00)) was significantly higher than for cohort studies (0.80 (0.66–0.94)). The sensitivity and specificity of presepsin obtained at a cutoff level of 722 μg/L was higher than its sensitivity and specificity at a cutoff level of 539 μg/L.

In addition, we performed a study of the appropriate cutoff interval (Additional file 1: Table S9). For PCT, 0.5–2 ng/mL may be the appropriate cutoff interval. Moreover, the 0.5–1 ng/mL PCT range had high sensitivity (0.88 (0.82–0.95)), whereas the 1.5–2 ng/mL range had high specificity (0.90 (0.77–1.00)). For CRP, ae cutoff value > 10 mg/L had high sensitivity (0.85 (0.72–0.98)) and specificity (0.93 (0.82–1.00)).

## Discussion

The main finding of this meta-analysis was that presepsin or PCT plus CRP improves the accuracy of diagnosis of neonatal sepsis. In addition, our meta-analysis initially established suitable cutoff values and cutoff intervals. However, there is significant statistical heterogeneity in some of the analyses. This fact cannot be ignored in the interpretation of our findings.

Significant differences in the definition of neonatal sepsis were observed in the studies included in our meta-analysis. Although the concept of neonatal clinical sepsis is widely used, a standard definition for this common condition has not been established [11], thereby resulting in the variability in the criteria that are used for diagnoses [12]. Therefore, it is likely that the term *neonatal sepsis* encompasses diseases and disease severity that differ among the studies included in this meta-analysis, and may also explain the high levels of heterogeneity observed in our analysis. In addition, the potential source of heterogeneity may be the age of the patients, so we have removed studies in patients older than 28 days. Inclusion of preterm neonates in evaluation studies may also be a source of heterogeneity. However, because the percentage of neonates born prematurely is rarely reported in studies, we were unable to assess the impact of this particular factor on the diagnosis of neonatal sepsis using biomarkers. In infants with low birth weight, infection is more common than in those with normal birth weight. One of the important risk factors is early rupture of the membrane (PROM), which may pose a risk of upward infection to the fetus (microorganisms in the external genitalia cause infection of the internal genitalia by ascending) [13]. In addition, local microbiological characteristics may influence the value of PCT in predicting neonatal sepsis [14, 15]. However, we cannot explore this further because the studies included in this meta-analysis do not provide information on microbiological characteristics.

An important advantage of using biomarkers in screening for neonatal sepsis is the ability to correctly identify neonates with culture-negative sepsis, who require antibiotic therapy [12]. In addition, it is also important to exclude the diagnosis of sepsis so that the number of neonates treated with antibiotics can be minimized, hospital stays can be shortened, selection pressure for resistant strains may appear to be smaller, and medical and economic advantages may offset the financial costs of measuring PCT (the cost of measuring CRP is approximately 25% of the cost of measuring PCT) [16]. We observed that although PCT is more sensitive than CRP, the use of PCT or CRP alone cannot rule out a diagnosis of neonatal sepsis. The combination of CRP and PCT resulted in higher sensitivity and AUC and lower NLR, which helped in confirming and ruling out neonatal sepsis. Therefore, it is important to combine these two biomarkers for the diagnosis of neonatal sepsis. In addition, this meta-analysis shows that presepsin may be used alone to diagnose and rule out neonatal sepsis due to its high sensitivity and specificity. Many studies have found that the level of presepsin in patients with sepsis is significantly higher than in healthy infants, and over time, similar to CRP and PCT, it decreases with antibiotic treatment [17–22]. These findings indicate that presepsin can be used to monitor clinical response to therapeutic interventions prior to obtaining culture results. Poggi et al. reported that even on the first day of treatment, the level of presepsin decreased, and CRP and PCT did not differ from the baseline values, suggesting that presepsin may be able to detect neonatal sepsis earlier than PCT or CRP [17]. Moreover, some studies found no correlation between presepsin levels and gestational age in the control group, and it appears that presepsin is not affected by postnatal age [17, 18]. Therefore, a unique presepsin reference range can be used for preterm or term infants on any day after birth. However, the results of this meta-analysis are based on prospective and retrospective studies, which have relatively different methodological characteristics. Subgroup analysis showed that the sensitivity of presepsin in the diagnosis of neonatal sepsis was significantly different between prospective and retrospective studies, suggesting that different study designs may influence the accuracy of the diagnostic trial. In addition, these studies did not use a predetermined cutoff value, but used an optimal cutoff value instead, which could lead to overestimation of diagnostic accuracy [17–22]. In some studies, the inclusion of too many healthy newborns in the control group also improved the diagnostic accuracy, so we excluded these studies. Overall, there are too few high-quality studies on presepsin, and more research is needed to support its use in the diagnosis and exclusion of neonatal sepsis.

Our meta-analysis has several limitations. First, we observed heterogeneity between the studies included, in the selection of neonatal features and the broad definition of neonatal sepsis. Some studies only confirm septicemia by positive blood cultures, microscopy, or polymerase chain reaction, whereas others also consider a comprehensive assessment of the patient chart and assessment of clinical, radiological, and laboratory data [23, 24]. Second, some studies did not provide the exact time of blood sampling, only indicating “on admission” or “before antibiotic treatment” [25, 26]. Third, previous studies have shown that PCT has better diagnostic accuracy in the diagnosis of late-onset sepsis [27], but the current data on early-onset and late-onset sepsis in this meta-analysis is not sufficient to generate any definitive conclusions. Fourth, we consider that differences in diagnostic accuracy in different regions may be due to a variety of reasons, such as individual patient differences, diagnostic criteria for neonatal sepsis, methods for detecting samples, laboratory testing levels, and instruments used. Since these studies did not provide the aforementioned details, we could not further analyze the specific causes of differences in diagnostic accuracy in different regions.

## Conclusions

The combination of PCT and CRP or presepsin alone improves the accuracy of the diagnosis of neonatal sepsis. However, further studies are required to confirm these findings.

## Additional files

Additional file 1: **Table S1.** The characteristics of the studies included. **Table S2.** The characteristics of the studies included. **Table S3.** Pair-wise comparisons between modalities for sensitivity, specificity, PLR, NLR, and AUC. **Table S4.** The result of meta-regression and subgroup analysis for PCT. **Table S5.** The result of meta-regression and subgroup analysis for CRP. **Table S6.** The result of meta-regression and subgroup analysis for presepsin. **Table S7.** Subgroup analysis of region and detection method for PCT and CRP. **Table S8.** Subgroup analysis of region and cutoff level for PCT and CRP. **Table S9.** Subgroup analysis of cutoff level for PCT and CRP. **Table S10.** Sensitivity analyses of PCT, CRP, PCT + CRP, and presepsin. (ZIP 100 kb)

Additional file 2: **Figure S1.** Bivariate boxplots. Bivariate boxplots of CRP (A), PCT (B), PCT plus CRP (C), and presepsin (D). (TIF 2191 kb)

Additional file 3: **Figure S2.** Likelihood ratio scattergrams. Scattergrams evaluating the positive likelihood ratios in the diagnosis of neonatal sepsis for CRP (A), PCT (B), PCT plus CRP (C), and presepsin (D). (TIF 2591 kb)

Additional file 4: **Figure S3.** Deek’s funnel plots. Funnel plots evaluating publication bias of CRP (A), PCT (B), PCT plus CRP (C), and presepsin (D). (TIF 1861 kb)

Additional file 5: **Figure S4.** Fagan diagram. A, Fagan diagram of CRP. B, Fagan diagram of PCT. C, Fagan diagram of PCT plus CRP. D, Fagan diagram of Presepsin. (TIF 3370 kb)

## Abbreviations

95% CI: Corresponding 95% credible interval
AUC: Area under the curve
CRP: C-reactive protein
DOR: Diagnostic odds ratio
FN: False negative
FP: False positive
NICU: Neonatal intensive care unit
NLR: Negative likelihood ratio
PCT: Procalcitonin
PLR: Positive likelihood ratio
QUADAS-2: Quality Assessment of Diagnostic Accuracy Studies
SROC: Summary receiver-operating characteristic
TN: True negative
TP: True positive
