# Supplementary material for: The combination of procalcitonin and C-reactive protein or presepsin alone improves the accuracy of diagnosis of neonatal sepsis: a meta-analysis and systematic review
Source: Crit Care. 2018 Nov 21;22:316. doi: 10.1186/s13054-018-2236-1 (PMC6249912; doi:10.1186/s13054-018-2236-1)
Supplement: Supplementary file 1 — Table S1. The characteristics of the studies included. Table S2. The characteristics of the studies included. Table S3. Pair-wise comparisons between modalities for sensitivity, specificity, PLR, NLR, and AUC. Table S4. The result of meta-regression and subgroup analysis for PCT. Table S5. The result of meta-regression and subgroup analysis for CRP. Table S6. The result of meta-regression and subgroup analysis for presepsin. Table S7. Subgroup analysis of region and detection method for PCT and CRP. Table S8. Subgroup analysis of region and cutoff level for PCT and CRP. Table S9. Subgroup analysis of cutoff level for PCT and CRP. Table S10. Sensitivity analyses of PCT, CRP, PCT + CRP, and presepsin. (ZIP 100 kb) [file 13054_2018_2236_MOESM1_ESM.zip › Supplement 3..docx]

**Table1: The characteristics of the included studies**

| **Study,year** | **Design** | **Region** | **Assay method** | | | **Cutoff** | | | **Patients(n)** | | |
| --- | --- | --- | --- | --- | --- | --- | --- | --- | --- | --- | --- |
|  |  |  | **PCT** | **CRP** | **Presepsin** | **PCT**  **(ng/mL)** | **CRP**  **(mg/L)** | **Presepsin**  **(ug/L)** | **Sep** | **Non-**  **sep** | **total** |
| Enguix et al, 2001 [18] | Case-control study | Europe | Immunoluminometric | Immunonephelometry | NA | 6.1 | 23 | NA | 20 | 26 | 46 |
| Franz et al, 1999 [19] | Cohort study | Europe | Immunoluminometric | chemiluminescent immunoassay | NA | 0.5 | 10 | NA | 46 | 116 | 162 |
| Blommendahl et al, 2002 [20] | Cohort study | Europe | immunoluminometric | Immunonephelometry | NA | 1 | 1 | NA | 13 | 156 | 169 |
| Chiesa et al, 2003 [1] | Cohort study | Europe | immunoluminometric | Immunonephelometry | NA | 1 | 4 | NA | 19 | 115 | 134 |
| Ko¨ksal et al, 2007 [21] | Cohort study | Asia | immunoluminometric | Immunonephelometry | NA | 1 | 10 | NA | 49 | 18 | 67 |
| Boo et al, 2008 [6] | Cohort study | Asia | Semi-quantitative PCT-Q | immunonephelometry | NA | 2 | NA | NA | 18 | 69 | 87 |
| Al-Zahrani, 2015 [22] | Cohort study | Africa | Enzyme immune assay | Enzyme immune assay | NA | 1.7 | 2.5 | NA | 71 | 29 | 100 |
| C¸etinkaya, 2009 [8] | Cohort study | Asia | immunoluminometric | Immunonephelometry | NA | 0.5 | 5 | NA | 123 | 40 | 163 |
| Groselj-Grenc, 2009 [23] | Cohort study | Europe | immunoluminometric | Immunonephelometry | NA | 2.28 | 11 | NA | 17 | 29 | 46 |
| Abdollahi et al, 2012 [7] | Cross-sectional study | Asia | Semi-quantitative PCT-Q | ELISA | NA | 4.7 | 8 | NA | 49 | 16 | 65 |
| Yuan et al, 2017 [24] | Case-control study | Asia | ECLIA | Immunonephelometry | NA | 1.53 | 13.5 | NA | 81 | 83 | 164 |
| Li et al, 2012 [25] | Case-control study | Asia | chemiluminescence | Immunonephelometry | NA | 2 | 10 | NA | 87 | 38 | 125 |
| Chen et al, 2013 [26] | Case-control study | Asia | Semi-quantitative PCT-Q | Immunonephelometry | NA | 0.5 | 10 | NA | 58 | 39 | 97 |
| LI et al,2014 [27] | Case-control study | Asia | immunoluminometric | Immunonephelometry | NA | 0.5 | 8 | NA | 50 | 50 | 100 |
| You et al, 2015 [28] | Case-control study | Asia | ECLIA | Immunonephelometry | NA | 0.5 | 8 | NA | 56 | 50 | 106 |
| Qin et al, 2012 [29] | Case-control study | Asia | Semi-quantitative PCT-Q | Immunonephelometry | NA | 0.5 | 8 | NA | 43 | 31 | 74 |
| Wei et al, 2015 [30] | Case-control study | Asia | ECLIA | Immunonephelometry | NA | 2 | 10 | NA | 50 | 50 | 100 |
| Guo et al, 2014 [31] | Case-control study | Asia | chemiluminescence | Immunonephelometry | NA | 0.1 | 3 | NA | 50 | 50 | 100 |
| Ma et al, 2012 [32] | Case-control study | Asia | ELFA | Immunonephelometry | NA | 0.5 | 5 | NA | 32 | 71 | 103 |
| Jacquot et al,2009 [33] | Cohort study | Europe | TRACE | Immunonephelometry | NA | 0.6 | 10 | NA | 30 | 43 | 73 |
| Vazzalwar et al,2005 [34] | Cohort study | North America | immunoluminometric | Immunonephelometry | NA | 0.5 | 8 | NA | 36 | 31 | 67 |
| Naher et al,2011 [13] | Cross-sectional study | Asia | Semi-quantitative PCT-Q | latex serology | NA | 0.5 | 6 | NA | 40 | 10 | 50 |
| Chiara Poggi et al,2015 [35] | Cohort study | Europe | NA | NA | CLEIA | NA | NA | 885 | 19 | 21 | 40 |
| Montaldo et al,2017 [36] | Case-control | Europe | NA | NA | CLEIA | NA | NA | 788 | 32 | 38 | 70 |
| Jehan H Sabry et al,2016 [37] | Case-control | Africa | NA | NA | ELISA | NA | NA | 722 | 80 | 40 | 120 |
| Ozdemir et al,2016 [38] | Cohort study | Asia | NA | NA | CLEIA | NA | NA | 539 | 29 | 40 | 69 |
| Topcuoglu et al,2016 [39] | Cohort study | Asia | NA | NA | CLEIA | NA | NA | 800.5 | 42 | 40 | 82 |
| Neeraj Kumar et al,2018 [40] | cross-sectional study | Asia | NA | NA | ELISA | NA | NA | 1800 | 41 | 41 | 82 |
| Total |  |  |  |  |  |  |  |  | 1281 | 1380 | 2661 |
|  |  |  |  |  |  |  |  |  |  |  |  |

ELISA: enzyme linked immunosorbent assay; ECLIA: electro-chemiluminescence Immunoassay assay; CLEIA: chemiluminescent enzyme immunoassay; ELFA:enzyme-linked fluorescent assay; TRACE: time-resolved amplified cryptate emission; TP=true positive; FP: false positive; FN: false negative; TN=true negative;NA:NOT Available

**Table 3: Pair-wise comparisons between modalities for sensitivity, Specificity, PLR, NLR, and AUC**

| **Category** | **Sensitivity** | **P** | **Specificity** | **p** | **PLR** | **p** | **NLR** | **p** | **AUC** | **p** |
| --- | --- | --- | --- | --- | --- | --- | --- | --- | --- | --- |
| PCT | 0.85 [0.79, 0.89] | NA | 0.84 [0.78, 0.89] | NA | 5.4 [3.7, 7.9] | NA | 0.18 [0.13, 0.25] | NA | 0.91 [0.89 - 0.94] | NA |
| CRP | 0.71 [0.63, 0.78] | NA | 0.88 [0.80, 0.93] | NA | 6.1 [3.6, 10.5] | NA | 0.33 [0.26, 0.42] | NA | 0.85 [0.82 - 0.88] | NA |
| PCT+CRP | 0.91 [0.84, 0.95] | NA | 0.89 [0.81, 0.93] | NA | 8.0 [4.6, 14.0] | NA | 0.10 [0.05, 0.19] | NA | 0.96 [0.93 - 0.97] | NA |
| Presepsin | 0.94 [0.80, 0.99] | NA | 0.98 [0.87, 1.00] | NA | 50.8 [6.5,394.7] | NA | 0.06 [0.02, 0.23] | NA | 0.99 [0.98 - 1.00] | NA |
| PCT vs CRP | **85% vs 71%** | **＜0.01✝** | 84% vs 88% | ＞0.05 | 5.4 vs 6.1 | ＞0.05 | 0.18 vs 0.33 | ＞0.05 | 0.91 vs 0.85 | 0.0192 |
| PCT+CRP vs PCT | 91% vs 85% | ＞0.05 | 89% vs 84% | ＞0.05 | 8.0 vs 5.4 | ＞0.05 | 0.10 vs 0.18 | ＞0.05 | 0.96 vs 0.91 | ＜0.05 |
| PCT+CRP vs CRP | **91% vs 71%** | **＜0.01✝** | 89% vs 88% | ＞0.05 | 8.0 vs 6.1 | ＞0.05 | **0.10 vs 0.33** | **＜0.01✝** | **0.96 vs 0.85** | **＜0.01✝** |
| PCT vs Presepsin | 85% vs 94% | ＞0.05 | 84% vs 98% | ＞0.05 | 5.4 vs 50.8 | ＞0.05 | 0.18 vs 0.06 | ＞0.05 | **0.91 vs 0.99** | **＜0.01✝** |
| CRP vs Presepsin | **71% vs 94%** | **＜0.01✝** | 88% vs 98% | ＞0.05 | 6.1 vs 50.8 | ＞0.05 | **0.33 vs 0.06** | **＜0.01✝** | **0.85 vs 0.99** | **＜0.01✝** |
| PCT+CRP vs Presepsin | 91% vs 94% | ＞0.05 | 89% vs 98% | ＞0.05 | 8.0 vs 50.8 | ＞0.05 | 0.10 vs 0.06 | ＞0.05 | **0.96 vs 0.99** | **＜0.01✝** |
|  |  |  |  |  |  |  |  |  |  |  |

PCT: procalcitonin; CRP:C-reactive protein; AUC: Area Under Curve PLR:positive likelihood ratio; NLR: negative likelihood ratio ✝significant, p<0.01

**Table 4: The result of meta-regression and subgroup analysis for PCT**

| **Category** | **NO.of Trails** | **Sensitivity(95%CI)** | **Specificity(95%CI)** | **I^2^(%)** | **P** |
| --- | --- | --- | --- | --- | --- |
| **region** |  |  |  |  |  |
| Africa | 1 | 0.71 [0.38 - 1.00] | 0.94 [0.81 - 1.00] | 0 | 0.39 |
| Asia | 14 | 0.85 [0.80 - 0.91] | 0.87 [0.81 - 0.93] | 11 | 0.32 |
| Europe | 6 | 0.85 [0.74 - 0.95] | 0.74 [0.61 - 0.88] | 47 | 0.15 |
| North America | 1 | 0.98 [0.92 - 1.00] | 0.81 [0.51 - 1.00] | 34 | 0.22 |
|  |  |  |  |  |  |
| **method** |  |  |  |  |  |
| ECLIA | 3 | 0.90 [0.82 - 0.99] | 0.81 [0.64 - 0.98] | 0 | 0.45 |
| ELFA | 1 | 0.94 [0.84 - 1.00] | 0.91 [0.74 - 1.00] | 0 | 0.42 |
| Enzyme immune assay | 1 | 0.71 [0.39 - 1.00] | 0.94 [0.81 - 1.00] | 0 | 0.38 |
| Immunoluminometric | 9 | 0.81 [0.72 - 0.90] | 0.85 [0.77 - 0.94] | 0 | 0.50 |
| Semi-quantitative PCT-Q | 5 | 0.84 [0.74 - 0.95] | 0.84 [0.71 - 0.97] | 0 | 0.99 |
| TRACE | 1 | 1.00 [0.99 - 1.00] | 0.65 [0.24 - 1.00] | 48 | 0.15 |
| chemiluminescence | 2 | 0.84 [0.68 - 0.99] | 0.85 [0.66 - 1.00] | 0 | 0.99 |
|  |  |  |  |  |  |
| **Test time** |  |  |  |  |  |
| 0 | 12 | 0.84 [0.77 - 0.91] | 0.87 [0.80 - 0.94] | 0 | 0.51 |
| 12 | 1 | 0.72 [0.39 - 1.00] | 0.83 [0.51 - 1.00] | 0 | 0.61 |
| 24 | 2 | 0.80 [0.60 - 1.00] | 0.82 [0.61 - 1.00] | 0 | 0.81 |
| Other (NA) | 7 | 0.89 [0.82 - 0.95] | 0.80 [0.69 - 0.91] | 10 | 0.33 |
|  |  |  |  |  |  |
| **design** |  |  |  |  |  |
| Case-control study | 10 | 0.90 [0.86 - 0.94] | 0.87 [0.80 - 0.94] | **76** | **0.02** |
| Cohort study | 10 | 0.81 [0.72 - 0.89] | 0.80 [0.71 - 0.90] | 42 | 0.18 |
| Cross-sectional study | 2 | 0.69 [0.45 - 0.92] | 0.87 [0.67 - 1.00] | 32 | 0.23 |
|  |  |  |  |  |  |
| **Cut-off** |  |  |  |  |  |
| 0.1 | 1 | 0.76 [0.48 - 1.00] | 0.89 [0.69 - 1.00] | 0 | 0.76 |
| 0.5 | 9 | 0.86 [0.79 - 0.93] | 0.90 [0.83 - 0.96] | 46 | 0.16 |
| 0.6 | 1 | 1.00 [0.99 - 1.00] | 0.65 [0.24 - 1.00] | 48 | 0.15 |
| 1 | 3 | 0.72 [0.52 - 0.91] | 0.86 [0.72 - 1.00] | 24 | 0.27 |
| 1.53 | 1 | 0.91 [0.77 - 1.00] | 0.86 [0.63 - 1.00] | 0 | 0.77 |
| 1.7 | 1 | 0.71 [0.39 - 1.00] | 0.94 [0.81 - 1.00] | 0 | 0.38 |
| 2 | 3 | 0.88 [0.78 - 0.99] | 0.69 [0.48 - 0.91] | 41 | 0.19 |
| 2.28 | 1 | 0.83 [0.56 - 1.00] | 0.48 [0.04 - 0.92] | 46 | 0.16 |
| 4.7 | 1 | 0.72 [0.40 - 1.00] | 0.83 [0.51 - 1.00] | 0 | 0.60 |
| 6.1 | 1 | 0.96 [0.85 - 1.00] | 0.89 [0.69 - 1.00] | 0 | 0.44 |
|  |  |  |  |  |  |

ECLIA: electro-chemiluminescence Immunoassay assay; ELFA:enzyme-linked fluorescent assay;PCT: procalcitonin;Significant results are underlined

**Table 5: The result of meta-regression and subgroup analysis for CRP**

| **Category** | **NO.of Trails** | **Sensitivity(95%CI)** | **Specificity(95%CI)** | **I^2^(%)** | **P** |
| --- | --- | --- | --- | --- | --- |
| **region** |  |  |  |  |  |
| Africa | 1 | 0.92 [0.80 - 1.00] | 0.73 [0.21 - 1.00] | 48 | 0.15 |
| Asia | 14 | 0.72 [0.63 - 0.80] | 0.87 [0.78 - 0.96] | 0 | 0.81 |
| Europe | 6 | 0.63 [0.47 - 0.79] | 0.92 [0.83 - 1.00] | 0 | 0.43 |
| North America | 1 | 0.73 [0.40 - 1.00] | 0.95 [0.79 - 1.00] | 0 | 0.82 |
|  |  |  |  |  |  |
| **method** |  |  |  |  |  |
| ELISA | 1 | 0.49 [0.12 - 0.86] | 1.00 [0.99 - 1.00] | 0 | 0.41 |
| Enzyme immune assay | 1 | 0.92 [0.80 - 1.00] | 0.73 [0.25 - 1.00] | 48 | 0.14 |
| Immunonephelometry | 18 | 0.73 [0.66 - 0.80] | 0.86 [0.78 - 0.93] | 25 | 0.26 |
| chemiluminescent immunoassay | 1 | 0.28 [0.01 - 0.55] | 0.98 [0.92 - 1.00] | **73** | **0.02** |
| latex serology | 1 | 0.55 [0.17 - 0.93] | 1.00 [0.99 - 1.00] | 0 | 0.66 |
|  |  |  |  |  |  |
| **Test time** |  |  |  |  |  |
| 0 | 12 | 0.71 [0.61 - 0.81] | 0.92 [0.86 - 0.98] | 40 | 0.19 |
| 12 | 1 | 0.49 [0.12 - 0.86] | 1.00 [0.99 - 1.00] | 0 | 0.43 |
| 24 | 2 | 0.80 [0.61 - 0.99] | 0.83 [0.59 - 1.00] | 0 | 0.69 |
| Other(NA) | 7 | 0.71 [0.59 - 0.84] | 0.78 [0.62 - 0.93] | 48 | 0.15 |
|  |  |  |  |  |  |
| **design** |  |  |  |  |  |
| Case-control study | 10 | 0.80 [0.73 - 0.87] | 0.78 [0.66 - 0.90] | **83** | **0.00** |
| Cohort study | 10 | 0.64 [0.53 - 0.75] | 0.92 [0.87 - 0.98] | **58** | 0.09 |
| Cross-sectional study | 2 | 0.52 [0.26 - 0.78] | 1.00 [0.99 - 1.00] | 46 | 0.15 |
|  |  |  |  |  |  |
| **Cut-off** |  |  |  |  |  |
| 1 | 1 | 0.62 [0.19 - 1.00] | 0.84 [0.50 - 1.00] | 0 | 0.83 |
| 2.5 | 1 | 0.92 [0.80 - 1.00] | 0.73 [0.24 - 1.00] | 48 | 0.15 |
| 3 | 1 | 0.80 [0.55 - 1.00] | 0.64 [0.09 - 1.00] | 0 | 0.49 |
| 4 | 1 | 0.74 [0.41 - 1.00] | 0.83 [0.48 - 1.00] | 0 | 0.94 |
| 5 | 2 | 0.77 [0.56 - 0.97] | 0.94 [0.70 - 1.00] | 0 | 0.76 |
| 6 | 1 | 0.55 [0.17 - 0.93] | 1.00 [0.99 - 1.00] | 0 | 0.66 |
| 8 | 5 | 0.70 [0.55 - 0.85] | 0.90 [0.79 - 1.00] | 0 | 0.89 |
| 10 | 6 | 0.67 [0.52 - 0.83] | 0.94 [0.83 - 1.00] | 0 | 0.77 |
| 11 | 1 | 0.59 [0.17 - 1.00] | 1.00 [0.99 - 1.00] | 0 | 0.48 |
| 13.5 | 1 | 0.89 [0.75 - 1.00] | 0.85 [0.54 - 1.00] | 25 | 0.26 |
| 23 | 1 | 0.96 [0.85 - 1.00] | 0.86 [0.54 - 1.00] | 50 | 0.14 |

ELISA: enzyme linked immunosorbent assay; PCT: procalcitonin;Significant results are underlined

**Table 6: The result of meta-regression and subgroup analysis for Presepsin**

| **Category** | **NO.of studies** | **Sensitivity(95%CI)** | **Specificity(95%CI)** | **I^2^(%)** | **P** |
| --- | --- | --- | --- | --- | --- |
| **region** |  |  |  |  |  |
| Africa | 1 | 1.00 [0.95 - 1.00] | 0.98 [0.87 - 1.00] | **66** | **0.04** |
| Asia | 3 | 0.81 [0.73 - 0.88] | **0.90 [0.93 - 0.95]** | 35 | 0.15 |
| Europe | 2 | 0.94 [0.84 - 0.99] | **1.00 [0.94 - 1.00]** | 47 | 0.10 |
|  |  |  |  |  |  |
| **method** |  |  |  |  |  |
| ELISA | 2 | 0.99 [0.98 - 1.00] | 0.97 [0.90 - 1.00] | **69** | **0.04** |
| Chemiluminescent enzyme immunoassay | 4 | 0.85 [0.73 - 0.97] | 0.99 [0.95 - 1.00] | **69** | **0.04** |
|  |  |  |  |  |  |
| **design** |  |  |  |  |  |
| Case-control study | 2 | **0.99 [0.95 - 1.00]** | 0.99 [0.97 - 1.00] | 44 | 0.17 |
| Cohort study | 3 | **0.80 [0.66 - 0.94]** | 0.97 [0.90 - 1.00] | **67** | **0.05** |
| Cross-sectional study | 1 | 0.98 [0.92 - 1.00] | 0.96 [0.82 - 1.00] | 0 | 0.63 |
|  |  |  |  |  |  |
| **Cut-off** |  |  |  |  |  |
| 539 | 1 | **0.79 [0.60 - 0.92]** | **0.75 [0.59 - 0.87]** | 48 | 0.14 |
| 722 | 1 | **1.00 [0.95 - 1.00]** | **0.98 [0.87 - 1.00]** | 25 | 0.26 |
| 788 | 1 | 0.94 [0.79 - 0.99] | 1.00 [0.91 - 1.00] | **73** | **0.02** |
| 800.5 | 1 | 0.67 [0.50 - 0.80] | 1.00 [0.91 - 1.00] | 44 | 0.17 |
| 885 | 1 | 0.95 [0.74 - 1.00] | 1.00 [0.84 - 1.00] | 40 | 0.11 |
| 1800 | 1 | 0.98 [0.87 - 1.00] | 0.95 [0.83 - 0.99] | 0 | 0.63 |
|  |  |  |  |  |  |
| **On set of sepsis** |  |  |  |  |  |
| EOS | 2 | 0.89 [0.67 - 1.00] | 0.94 [0.79 - 1.00] | 0 | 0.41 |
| LOS | 3 | 0.95 [0.85 - 1.00] | 0.99 [0.98 - 1.00] | 8 | 0.34 |
| EOS+LOS | 1 | 0.98 [0.92 - 1.00] | 0.96 [0.82 - 1.00] | 0 | 0.63 |
|  |  |  |  |  |  |

**Table 7: Subgroup analysis of region and detection method for PCT and CRP**

| **Region** | **Dection** | **NO.of studies** | **Sensitivity(95%CI)** | **Specificity(95%CI)** |
| --- | --- | --- | --- | --- |
| **PCT** |  |  |  |  |
| Asia | ECLIA | 3 | 0.90 [0.82 - 0.99] | 0.81 [0.64 - 0.98] |
|  | ELFA | 1 | 0.94 [0.84 - 1.00] | 0.91 [0.74 - 1.00] |
|  | Immunoluminometric | 3 | **0.75 [0.60 - 0.89]** | 0.96 [0.91 - 1.00] |
|  | chemiluminescence | 2 | 0.84 [0.68 - 0.99] | 0.85 [0.66 - 1.00] |
|  | Semi-quantitative PCT-Q | 5 | 0.84 [0.74 - 0.95] | 0.84 [0.71 - 0.97] |
|  |  |  |  |  |
| Europe | Immunoluminometric | 5 | **0.78 [ 0.62 - 0.89]** | 0.77 [0.56 - 0.90] |
| Africa | Enzyme immune assay | 1 | 0.71 [0.39 - 1.00] | 0.94 [0.81 - 1.00] |
| North America | Immunoluminometric | 1 | **0.98 [0.92 - 1.00]** | 0.81 [0.51 - 1.00] |
|  |  |  |  |  |
| **CRP** |  |  |  |  |
| Asia | ELISA | 1 | 0.49 [0.12 - 0.86] | 1.00 [0.99 - 1.00] |
|  | Immunonephelometry | 12 | **0.74 [0.67 - 0.80]** | **0.84 [0.69 - 0.93]** |
|  | latex serology | 1 | 0.55 [0.17 - 0.93] | 1.00 [0.99 - 1.00] |
| Europe | Immunonephelometry | 5 | **0.71 [0.54 - 0.83]** | **0.85 [0.81 - 0.89]** |
|  | chemiluminescent immunoassay | 1 | 0.28 [0.01 - 0.54] | 0.98 [0.92 - 1.00] |
| Africa | Enzyme immune assay | 1 | 0.92 [0.80 - 1.00] | 0.73 [0.25 - 1.00] |
| North America | Immunonephelometry | 1 | **0.73 [0.40 - 1.00]** | 0.95 [0.79 - 1.00] |
|  |  |  |  |  |

ECLIA: electro-chemiluminescence Immunoassay assay; ELFA:enzyme-linked fluorescent assay; PCT: procalcitonin; Significant results are underline

**Table 8: Subgroup analysis of region and cutoff level for PCT and CRP**

| **Region** | **Cut-off (ng/ml)** | **NO.of studies** | **Sensitivity(95%CI)** | **Specificity(95%CI)** |
| --- | --- | --- | --- | --- |
| **PCT** |  |  |  |  |
| Asia | 0.1 | 1 | 0.76 [0.48 - 1.00] | 0.89 [0.69 - 1.00] |
|  | 0.5 | 7 | 0.88 [0.79 - 0.93] | 0.94 [0.87 - 0.97] |
|  | 1 | 1 | **0.59 [0.26 - 0.91]** | 0.89 [0.70 - 1.00] |
|  | 1.53 | 1 | **0.91 [0.77 - 1.00]** | 0.86 [0.63 - 1.00] |
|  | 2 | 3 | 0.88 [0.78 - 0.99] | 0.69 [0.48 - 0.91] |
|  | 4.7 | 1 | 0.72 [0.40 - 1.00] | 0.83 [0.51 - 1.00] |
| Europe | 0.5 | 1 | 0.57 [0.37 - 0.76] | 0.67 [0.25 - 1.00] |
|  | 1 | 2 | 0.79 [0.51 - 1.00] | 0.83 [0.68 - 0.99] |
|  | 2.28 | 1 | 0.83 [0.56 - 1.00] | 0.48 [0.04 - 0.92] |
|  | 6.1 | 1 | 0.96 [0.85 - 1.00] | 0.89 [0.69 - 1.00] |
| Africa | 1.7 | 1 | 0.71 [0.38 - 1.00] | 0.94 [0.81 - 1.00] |
| North America | 0.5 | 1 | 0.98 [0.92 - 1.00] | 0.81 [0.51 - 1.00] |
|  |  |  |  |  |
| **CRP** |  |  |  |  |
| Asia | 3 | 1 | 0.80 [0.55 - 1.00] | 0.64 [0.09 - 1.00] |
|  | 5 | 2 | 0.77 [0.56 - 0.97] | 0.94 [0.70 - 1.00] |
|  | 6 | 1 | 0.55 [0.17 - 0.93] | 1.00 [0.99 - 1.00] |
|  | 8 | 4 | 0.69 [0.56 - 0.82] | 0.89 [0.72 - 1.00] |
|  | 10 | 4 | 0.69 [0.56 - 0.82] | 0.85 [0.64 - 1.00] |
|  | 13.5 | 1 | 0.89 [0.75 - 1.00] | 0.85 [0.54 - 1.00] |
|  | NA | 1 | 0.56 [0.14 - 0.98] | 0.90 [0.68 - 1.00] |
| Europe | 1 | 1 | 0.62 [0.19 - 1.00] | 0.84 [0.50 - 1.00] |
|  | 4 | 1 | 0.74 [0.41 - 1.00] | 0.83 [0.48 - 1.00] |
|  | 10 | 2 | **0.41 [0.24 - 0.59]** | **0.94 [0.89 - 0.99]** |
|  | 11 | 1 | 0.59 [0.17 - 1.00] | **1.00 [0.99 - 1.00]** |
|  | 23 | 1 | **0.96 [0.85 - 1.00]** | 0.86 [0.54 - 1.00] |
| Africa | 2.5 | 1 | 0.92 [0.80 - 1.00] | 0.73 [0.24 - 1.00] |
| North America | 8 | 1 | 0.73 [0.40 - 1.00] | 0.95 [0.79 - 1.00] |

PCT: procalcitonin; CRP:C-reactive protein; Significant results are underlined.

**Table 9: Subgroup analysis of cut-off level for PCT and CRP**

| **Cut-off(ng/mL)** | **NO. of stuides** | **Summary statistics** | |
| --- | --- | --- | --- |
| **PCT** |  |  |  |
| **O.5-2** |  |  | SEN=0.86 [0.77 - 0.91] |
| **0.5-1** | 10 | SEC=0.88 [0.82 - 0.95] | SPE=0.88 [0.81 - 0.93] |
|  |  | SPE=0.88 [0.81 - 0.95] | PLR=7.0 [4.3 - 11.4] |
|  |  |  | NLR=0.16 [0.10 - 0.26] |
| **1-1.5** | 3 | SEC=0.72 [0.51 - 0.93] | DOR=43 [21 - 90] |
|  |  | SPE=0.86 [0.71 - 1.00] | **AUC= 0.93 [0.91 - 0.95]** |
|  |  |  |  |
| **1.5-2** | 2 | SEC=0.82 [0.65 - 1.00] |  |
|  |  | SPE=0.90 [0.77 - 1.00] |  |
|  |  |  |  |
| **2-2.5** | 4 | SEC=0.88 [0.77 - 0.98] |  |
|  |  | SPE=0.65 [0.46 - 0.83] |  |
|  |  | PLR=2.4 [1.8 - 3.2] |  |
|  |  | NLR=0.20 [0.13 - 0.32] |  |
|  |  | DOR=12 [6 - 23] |  |
|  |  | **AUC=0.87 [0.84 - 0.90]** |  |
|  |  |  |  |
| **＞2.5** | 2 | 0.84 [0.66 - 1.00] |  |
|  |  | 0.87 [0.68 - 1.00] |  |
| **CRP** |  |  |  |
| **≤6** | 7 | SEN=0.76 [0.65 - 0.88] |  |
|  |  | SPE=0.84 [0.70 - 0.99] |  |
|  |  |  |  |
| **8** | 5 | SEN=0.70 [0.55 - 0.85] |  |
|  |  | SPE=0.90 [0.79 - 1.00] |  |
|  |  |  |  |
| **10** | 6 | SEN=0.60 [0.46 - 0.74] |  |
|  |  | SPE=0.88 [0.76 - 1.00] |  |
|  |  |  |  |
| **＞10** | 3 | SEN=0.85 [0.72 - 0.98] |  |
|  |  | SPE=0.93 [0.82 - 1.00] |  |
|  |  |  |  |
|  |  |  |  |
|  |  |  |  |

SEN: sensitivity; SPE:specificity PLR:positive likelihood ratio; NLR: negative likelihood ratio; DOR:diagnostic odds ratio;AUC: area under the curve.

**Table 10: Sensitivity analyses of PCT, CRP, PCT+CRP, and Presepsin**

| **Study** | **DOR(PCT)** | **95%CI** | **DOR(CRP)** | **95%CI** | **DOR(PCT+CRP)** | **95%CI** | **DOR(Presepsin)** | **95%CI** |
| --- | --- | --- | --- | --- | --- | --- | --- | --- |
| Franz et al,1999 | 36 | [21, 60] | 19 | [10, 36] | 80 | [49, 287] | NA | NA |
| Blommendahl et al, 2002 | 34 | [19, 60] | 20 | [10, 39] | 79 | [27, 330] | NA | NA |
| Yuan et al,2017 | 30 | [17, 54] | 17 | [9, 32] | 75 | [20, 212] | NA | NA |
| Li et al,2012 | 31 | [17, 56] | 20 | [10, 39] | 80 | [27, 329] | NA | NA |
| Chen et al,2013 | 28 | [16, 49] | 16 | [9, 28] | 70 | [21, 239] | NA | NA |
| LI et al,2014 | 28 | [16, 50] | 17 | [9, 31] | 79 | [20, 174] | NA | NA |
| Qin et al,2012 | 30 | [17, 54] | 21 | [11, 39] | 75 | [21, 259] | NA | NA |
| Wei et al,2015 | 33 | [18, 59] | 21 | [11, 38] | 73 | [21, 252] | NA | NA |
| Guo et al,2014 | 31 | [17, 57] | 20 | [10, 39] | 73 | [21, 252] | NA | NA |
| Ma et al,2012 | 29 | [16, 51] | 20 | [10, 38] | NA | NA | NA | NA |
| Jacquot et al,2009 | 29 | [16, 52] | 20 | [10, 39] | NA | NA | NA | NA |
| Vazzalwar et al,2005 | 29 | [16, 53] | 18 | [9, 35] | NA | NA | NA | NA |
| Naher et al,2011 | 32 | [18, 57] | 18 | [10, 34] | NA | NA | NA | NA |
| Enguix et al,2001 | 29 | [16, 52] | 18 | [9, 35] | NA | NA | NA | NA |
| You et al,2015 | 29 | [16, 52] | 20 | [10, 39] | NA | NA | NA | NA |
| Chiesa et al,2003 | 29 | [16, 52] | 19 | [10, 38] | NA | NA | NA | NA |
| Ko¨ksal et al,2007 | 32 | [18, 57] | 20 | [10, 38] | NA | NA | NA | NA |
| Boo et al 2008 | 32 | [18, 58] | 19 | [10, 38] | NA | NA | NA | NA |
| Al-Zahrani,2015 | 31 | [17, 56] | 19 | [9, 36] | NA | NA | NA | NA |
| C¸etinkaya,2009 | 28 | [16, 51] | 16 | [9, 29] | NA | NA | NA | NA |
| Groselj-Grenc,2009 | 33 | [19, 59] | 17 | [9, 31] | NA | NA | NA | NA |
| Abdollahi et al,2012 | 33 | [18, 59] | 18 | [9, 34] | NA | NA | NA | NA |
| Chiara Poggi et al,2015 | NA | NA | NA | NA | NA | NA | 860 | [65, 11472] |
| Montaldo et al,2017 | NA | NA | NA | NA | NA | NA | 857 | [68, 11471] |
| Jehan H Sabry et al,2016 | NA | NA | NA | NA | NA | NA | 855 | [65, 11470] |
| Ozdemir et al,2016 | NA | NA | NA | NA | NA | NA | 868 | [67, 11473] |
| Topcuoglu et al,2016 | NA | NA | NA | NA | NA | NA | 866 | [66, 11475] |
| Neeraj Kumar et al,2018 | NA | NA | NA | NA | NA | NA | 864 | [66, 11478] |
| combined | 31 | [17, 54] | 19 | [10, 35] | 79 | [26, 246] | 864 | [65, 11473] |

DOR:diagnostic odds ratio; 95%CI:95% Confidence interval.
