# Supplementary material for: The combination of procalcitonin and C-reactive protein or presepsin alone improves the accuracy of diagnosis of neonatal sepsis: a meta-analysis and systematic review
Source: Crit Care. 2018 Nov 21;22:316. doi: 10.1186/s13054-018-2236-1 (PMC6249912; doi:10.1186/s13054-018-2236-1)
Supplement: Supplementary file 1 — Table S1. The characteristics of the studies included. Table S2. The characteristics of the studies included. Table S3. Pair-wise comparisons between modalities for sensitivity, specificity, PLR, NLR, and AUC. Table S4. The result of meta-regression and subgroup analysis for PCT. Table S5. The result of meta-regression and subgroup analysis for CRP. Table S6. The result of meta-regression and subgroup analysis for presepsin. Table S7. Subgroup analysis of region and detection method for PCT and CRP. Table S8. Subgroup analysis of region and cutoff level for PCT and CRP. Table S9. Subgroup analysis of cutoff level for PCT and CRP. Table S10. Sensitivity analyses of PCT, CRP, PCT + CRP, and presepsin. (ZIP 100 kb) [file 13054_2018_2236_MOESM1_ESM.zip › Supplement 1.docx]

Supplement

Supplement 1. The Search strategy for pubmed

Supplement 2. Table 2

Supplement 3. Tables 1-14(exclude Table 2)

#1 ((((((((((((((("Calcitonin"[Mesh]) OR Calcitrin[Title/Abstract]) OR Thyrocalcitonin[Title/Abstract]) OR Calcitonin(1-32)[Title/Abstract]) OR Pro-Calcitonin[Title/Abstract]) OR Pro Calcitonin[Title/Abstract]) OR Calcitonin-1[Title/Abstract]) OR Calcitonin 1[Title/Abstract]) OR Calcitonin Related Polypeptide Alpha[Title/Abstract]) OR Calcitonin Precursor Polyprotein[Title/Abstract]) OR Procalcitonin[Title/Abstract]) OR Eel Calcitonin[Title/Abstract]) OR Calcitonin, Eel[Title/Abstract]) OR Ciba 47175-BA[Title/Abstract]) OR Ciba 47175 BA[Title/Abstract]) OR Ciba 47175BA[Title/Abstract]

#2 ((("C-Reactive Protein"[Mesh]) OR C Reactive Protein[Title/Abstract]) OR Protein, C-Reactive[Title/Abstract])

#3 (((("presepsin protein, human" [Supplementary Concept]) OR Peptide Fragments[Title/Abstract]) OR Lipopolysaccharide Receptors[Title/Abstract]) OR CD14[Title/Abstract]) OR sCD14-ST[Title/Abstract]

#4 ((((((((((((((((("Neonatal Sepsis"[Mesh]) OR Neonatal Sepses[Title/Abstract]) OR Sepses, Neonatal[Title/Abstract]) OR Sepsis, Neonatal[Title/Abstract]) OR Neonatal Late-Onset Sepsis[Title/Abstract]) OR Late-Onset Sepses, Neonatal[Title/Abstract]) OR Late-Onset Sepsis, Neonatal[Title/Abstract]) OR Neonatal Late Onset Sepsis[Title/Abstract]) OR Neonatal Late-Onset Sepses[Title/Abstract]) OR Sepses, Neonatal Late-Onset[Title/Abstract]) OR Sepsis, Neonatal Late-Onset[Title/Abstract]) OR Neonatal Early-Onset Sepsis[Title/Abstract]) OR Early-Onset Sepses, Neonatal[Title/Abstract]) OR Early-Onset Sepsis, Neonatal[Title/Abstract]) OR Neonatal Early Onset Sepsis[Title/Abstract]) OR Neonatal Early-Onset Sepses[Title/Abstract]) OR Sepses, Neonatal Early-Onset[Title/Abstract]) OR Sepsis, Neonatal Early-Onset[Title/Abstract]

#5 #1 OR # 2 OR #3

#6 #5 AND #4
