# Supplementary material for: The combination of procalcitonin and C-reactive protein or presepsin alone improves the accuracy of diagnosis of neonatal sepsis: a meta-analysis and systematic review
Source: Crit Care. 2018 Nov 21;22:316. doi: 10.1186/s13054-018-2236-1 (PMC6249912; doi:10.1186/s13054-018-2236-1)
Supplement: Supplementary file 1 — Table S1. The characteristics of the studies included. Table S2. The characteristics of the studies included. Table S3. Pair-wise comparisons between modalities for sensitivity, specificity, PLR, NLR, and AUC. Table S4. The result of meta-regression and subgroup analysis for PCT. Table S5. The result of meta-regression and subgroup analysis for CRP. Table S6. The result of meta-regression and subgroup analysis for presepsin. Table S7. Subgroup analysis of region and detection method for PCT and CRP. Table S8. Subgroup analysis of region and cutoff level for PCT and CRP. Table S9. Subgroup analysis of cutoff level for PCT and CRP. Table S10. Sensitivity analyses of PCT, CRP, PCT + CRP, and presepsin. (ZIP 100 kb) [file 13054_2018_2236_MOESM1_ESM.zip › Supplement 2.docx]

**Table 2: The characteristics of the included studies**

| **Author,**  **publication year** | **Country** | **Study period** | **Study design** | **Study population** | | | **Sepsis onset** | **Characteristics and number of patients** | |  |
| --- | --- | --- | --- | --- | --- | --- | --- | --- | --- | --- |
|  |  |  |  | **Age(day)**  **(sep/Non-sep)** | **Gestational**  **age(sep/Non-sep)(weeks)**  **(mean ± SD)** | **Weight(sep/**  **Non-sep) (mean ± SD, g)** |  | **Septic group** | **Non-septic group** | |
| Enguix et al,2001 [18] | spain | NA | Case control study | 3-30 | NA | ＜2500g : n=2 | NA | SIRS and positive blood culture or meningococcal rash or recovery with antibiotics: n = 20 | Negative infectious status: n = 26 | |
| Yang et al,2015  (excluded) | China | July,2013 to Dec,2014 | Case control study | 17.93±13.31/ 18.44±3.87 | 34.3±1.8/ 37.5±1.6 | 2500±300/  2900±330 | NA | Positive blood culture and the clinical features and symptoms suggestive of clinical sepsis:n=60 | They were not suspected of being clinically septic and had normal physical examination findings: n=60 | |
| Franz et al,1999 [19] | Germany | June,1997 to Feb ,1998 | Prospective cohort  study | NA | 33±9 52 infants <30 weeks | NA | NA | Culture-proved BI (≥1 clinical sign compatible with BI and positive blood culture): n = 9  Clinical BI (≥1 clinical sign compatible with BI and a CRP[>10 mg/l at 12–60 h after the first blood sample was taken): n = 37 | No evidence of BI: n = 116 | |
| Blommendahl et al, 2002 [20] | Finland | June,1997 to January,1999 | Prospective cohort  study | NA | 37.7 | 3090 | NA | Positive blood culture and clinical symptoms: n = 13  Negative blood culture and clinical symptoms: n = 156 | NA | |
| Chiesa et al,2003 [1] | Italy | July,2001 to Dec, 2001 | Prospective cohort  study | NA | 33.8 ± 4.3 | 2153 ± 903 | EOS:19 | Blood culture positive and definite, persistent clinical signs of sepsis prompting ≥5 days of antibiotic treatment: n = 11  Blood culture negative and definite, persistent clinical signs of sepsis prompting ≥5 days of antibiotic treatment: n = 8  Uncertain (systemic infection could be neither confirmed nor excluded): n = 20 | Symptomatic babies with negative  body fluid cultures, apparently well within 24–48h and had a benign clinical course until discharge and antibiotic treatment for ≤3 days: n = 115 | |

EOS: early onset sepsis, LOS: late-onset sepsis, BI: bacterial infection, SIRS:Systemic Inflammatory Response Syndrome

**Table 2 continued**

| **Author,**  **publication year** | **Country, Region** | **Study period** | **Study design** | **Study population** | | | **Sepsis onset** | **Characteristics and number of patients** | |  |
| --- | --- | --- | --- | --- | --- | --- | --- | --- | --- | --- |
|  |  |  |  | **Age(day)**  **(sep/Non-sep)** | **Gestational**  **age(sep/Non-sep)(weeks)**  **(mean ± SD)** | **Weight(sep/**  **Non-sep) (mean ± SD, g)** |  | **Septic group** | **Non-septic group** | |
| Ko¨ksal et al,2007  [21] | Turkey | Apr to Oct,2002 | Case control study | NA | 33.6 ± 4.8 | 1923 ± 841 | NA | High probable sepsis: (blood culture positive or negative, ≥3 sepsis-related clinical signs, CRP[>1mg/dl, and ≥2 additional altered serum parameters): n = 15  Probable sepsis: (blood culture negative, ≥3sepsis-related clinical signs, CRP[>1 mg/dl and ≥2 additional altered serum parameters): n = 14  Possible sepsis: (blood culture negative, <3 sepsis-related clinical signs, CRP<1 mg/dl, and<2 additional altered serum parameters): n = 20 | Negative blood culture, no sepsis related clinical signs, CRP<1 mg/dl, and no altered serum parameters: n = 18 | |
| Sakha et al,2008（excluded） | Iran | Apr,2007 to Apr,2008 | retrospective Cross-sectional | 11.12±8.94/ 7.99±8.01 | ＜34 | 2715.93±412.1/2871.89±640.64 | NA | Proven sepsis: (positive blood culture): n = 27 | Suspected sepsis: (negative blood  culture, positive CRP, and  neutropenia/  thrombocytopenia + chest  X-ray findings): n = 90 | |
| Boo et al 2008 [6] | Malaysia | Janu,2005 to  Dec, 2006 | prospective cohort  study | 0-30 | 30/34 | 1060/2100 | EOS and  LOS | Proven sepsis:(any symptomatic infants with positive blood culture results.): n = 18  a.12 had gram-positive sepsis  b. 6 had gram-negative sepsis | negative blood culture | |
| Al-Zahrani,2015 [22] | Egypt | Janu,2013-Janu,2014 | prospective cohort  study | NA | Preterm 18/2 | ＜2,500g: 28/9 | EOS:n=34  LOS:n=37 | Proven EOS: neonates with positive blood culture and/or positive PCR results for bacterial 16S rDNA: n=34  Clinical LOS: neonates with negative blood culture who had positive clinical signs consistent of sepsis, and positive sepsis screen based on band cells > 20%, polymorphocytosis, elevated CRP level: n=37 | negative infectious status or non-sepsis: neonates suspected of having sepsis who had negative blood culture and negative sepsis screen | |

EOS: early onset sepsis, LOS: late-onset sepsis, CRP: C-Reactive Protein

**Table 2 continued**

| **Author,**  **publication year** | **Country, Region** | **Study period** | **Study design** | **Study population** | | | **Sepsis onset** | **Characteristics and number of patients** | |  |
| --- | --- | --- | --- | --- | --- | --- | --- | --- | --- | --- |
|  |  |  |  | **Age(day)**  **(sep/Non-sep)** | **Gestational**  **age(sep/Non-sep)(weeks)**  **(mean ± SD)** | **Weight(sep/**  **Non-sep) (mean ± SD, g)** |  | **Septic group** | **Non-septic group** | |
| C¸etinkaya,2009  [8] | Turkey | Janu,2006  to Janu,2008 | Prospective cohort  study | 0-28 | 31.2 ± 3.19 | 1745 | Highly probable sepsis group: EOS,10/108  (9.3%),LOS, 98/108(90.7%).  Probable and possible sepsis groups:EOS,8/15(52.3%), LOS,7/15 (47.7%) | Highly probable sepsis: (blood culture positive or negative, ≥3 sepsis-related clinical signs, CRP[>1 mg/100 ml, and ≥2 additional altered serum parameters): n = 108  Probable sepsis: (blood culture negative, <3 sepsis-related clinical signs, CRP[>1 mg/100 ml, ≥2 additional altered serum parameters)] ＋[possible sepsis (blood culture negative, <3 sepsis-related clinical signs, CRP<1 mg/100 ml and <2 additional altered serum parameters): n = 15 | CRP <1 mg per 100 ml  No altered serum parameters  Negative blood culture, no sepsis related clinical signs: n = 40 | |
| Ertuğrul,2013  (excluded) | Turkey | Feb,2001 to May,2011 | Case control study | 12.54 ± 5.06 /10.35 ± 2.54 | 32.88 ± 1.45/  33 ± 1.49 | 1792±516.49/  1805 ± 506.39 | LOS | Babies diagnosed by clinical and laboratory findings as late neonatal sepsis. | healthy neonates | |
| Groselj-Grenc  et al,2009 [23] | Slovenia | NA | prospective cohort study | 1 (0–18)  0 (0–13) | 38.3(32.4–41.1) 38.4(32.4–41.4) | 3,050g (1,400–3,850) | Early and Late | SIRS and positive blood culture or meningococcal rash or recovery with antibiotics: n = 17 | Negative infectious status: n = 29 | |
| Kocabas et al，2007(excluded) | Turkey | May,2000 to Janu,2001 | Case control study | 6/5 | 38(35.8±4.10) 38（37.3±2.4） | 2938.1 ± 1137.3 | EOS: 13/26 (50%)  LOS: 13/26 (50%) | Sepsis patients: positive blood culture and clinical signs of sepsis: n = 26 | Healthy neonates: n = 29 | |
| Jacquot et al,2009  [33] | France | June,2005 to May,2006 | Prospective cohort  study | 11 (8-19)/  12 (8-18) | 28 (26–29)/  28 (26–31) | 990(705–1220)/1030(765–1460) | LOS | Infected patients: [definite (positive blood  culture/meningitis/pneumonia) or possible  infection (no pathogen identified)]: n = 30 | Non-infected patients: n = 43 | |
| Kwabena et al, 2017 (exclude) | Ghana | November 2013 to June 2014. | cross-sectional study | NA | NA | NA | EOS | Eligible neonates were categorized as ''highly probable'', ''probable'' and ''less probable'' neonatal sepsis based on an adapted criteria from Tollner's sepsis score and case definition of bloodstream infection by Vergnano et al. | NA | |

EOS: early onset sepsis, LOS: late-onset sepsis, CRP: C-Reactive Protein

Table 2 continued

| **Author,**  **publication year** | **Country, Region** | **Study period** | **Study design** | **Study population** | | | **Sepsis onset** | **Characteristics and number of patients** | |  |
| --- | --- | --- | --- | --- | --- | --- | --- | --- | --- | --- |
|  |  |  |  | **Age(day)**  **(sep/Non-sep)** | **Gestational**  **age(sep/Non-sep)(weeks)**  **(mean ± SD)** | **Weight(sep/**  **Non-sep) (mean ± SD, g)** |  | **Septic group** | **Non-septic group** | |
| Yuan et al,2017  [24] | China | Janu,2014-June,2015 | Case control study | 9/7 | 39 | 3316/3472 | EOS:34  LOS:47 | N=81   1. Confirm diagnosis*:   Clinical manifestations and meet any of the following: (1) blood culture or sterile body cavity culture of pathogens; (2) if the blood culture specimens cultivate pathogens, then must be with another (The same species of bacteria is cultured in blood, or in a sterile body lumen, or in a catheter tip).   1. Clinical diagnosis§: clinical manifestations and any of the following: 2. non-specific examination ≥ 2.   (2) positive blood test antigen or DNA test   1. Non-specific examination:(1)WBC decreased (<5×109/L), or WBC increased (≤3d WBC>25×109/L;>3d WBC>20×109/L).   (2)White blood cell classification: rod-shaped nucleus cells/neutrophils (immature/totalneutrophils, I/T) ≥ 0.16.  (3)C-reactive protein (CRP):≥8 μg/ml (peripheral blood method).  (4) Platelets ≤ 100 × 109/L.  (5)Erythrocyte sedimentation rate (ESR)≥15mm/h. | negative blood cultur:n=83 | |

EOS: early onset sepsis, LOS: late-onset sepsis, CRP: C-Reactive Protein

**Table 2 continued**

| **Author,**  **publication year** | **Country, Region** | **Study period** | **Study design** | **Study population** | | | **Sepsis onset** | **Characteristics and number of patients** | |  |
| --- | --- | --- | --- | --- | --- | --- | --- | --- | --- | --- |
|  |  |  |  | **Age(day)**  **(sep/Non-sep)** | **Gestational**  **age(sep/Non-sep)(weeks)**  **(mean ± SD)** | **Weight(sep/**  **Non-sep) (mean ± SD, g)** |  | **Septic group** | **Non-septic group** | |
| Gu et al,2015  (excluded) | China | Janu,2013-Feb,2014 | Retrospective case control study | 9.9±5.9/  9.4±6.5 | NA | NA | NA | N=54  1. Confirm diagnosis*   1. Clinical diagnosis§ | Healthy newborns and preterm infants without comorbidities:n=35 | |
| Li et al,2012 [25] | China | NA | case  control study | 0-21/3-23 | 34-42/  37-42 | 2 200-4000/  2 500-3800 | NA | N=58  1. Confirm diagnosis*: n=21  2. Clinical diagnosis§: n=37 | Non-septic group:n=20 | |
| Chen et al,2013 [26] | China | Janu-Dec 2012 | case  control study | 10/14.7 | NA | NA | NA | N=58  1. Confirm diagnosis*  2. Clinical diagnosis§ | Non-septic group:n=39 | |
| LI et al,2014 [27] | China | Janu-June,2013 | case  control study | 11.2±2.9/  11.3±2.7 | 39.0±1.6/38.9±1.7 | 3100.6±257.1/  3101.5±256.4 | EOS:22  LOS:28 | N=50  1. Confirm diagnosis*  2. Clinical diagnosis§ | Non-septic group:n=50 | |
| You et al,2015 [28] | China | May,2012-May,2014 | case  control study | 4-12/4-9 | Full-term:  n=46/36  pre-term: n=10/14 | 2230-4320/  2020-4150 | EOS:23  LOS:33 | N=56  1. Confirm diagnosis*  2. Clinical diagnosis§ | Non-septic group:n=50 | |
| Qin et al,2012 [29] | China | 2010-2011 | case  control study | NA | NA | NA | NA | N=43  1. Confirm diagnosis*:n=25  2. Clinical diagnosis§:n=18 | N=31  Non-sepsis group:  19 cases of local infection group 12 cases of viral infection. | |
| Liao et al,2013  （excluded） | China | Janu,2010-Apr,2012 | case  control study | 5. 9±3.7  6.2±3.5  6.1±3.6 | NA | NA | NA | N=95  1. Confirm diagnosis*  2. Clinical diagnosis§ | local infection: n=53  Healthy newborn:n= 40 | |
| Li Z,2012  (excluded） | China | Mar,2008-Aug,2010 | case  control study | 4.6±2.0/7.5±5.0 | NA | NA | NA | N=87  1. Confirm diagnosis*  2. Clinical diagnosis§ | Healthy newborn:n= 38 | |
| Xie J et al,2010  （excluded） | China | Janu,2008-June,2009 | case  control study | 3d | NA | NA | NA | N=80  1. Confirm diagnosis*  2. Clinical diagnosis§ | Healthy newborn:n= 50 | |
| Qiu et al,2015  （excluded） | China | Janu,2013-Dec,2014 | case  control study | 6.2±4.1/  5.8±3.7/  5.3±3.0 | NA | NA | NA | N=67  1. Confirm diagnosis*  2. Clinical diagnosis§ | local infection: n=63  Healthy newborn:n= 65 | |

EOS: early onset sepsis, LOS: late-onset sepsis

**Table 2 continued**

| **Author,**  **publication year** | **Country, Region** | **Study period** | **Study design** | **Study population** | | | **Sepsis onset** | **Characteristics and number of patients** | |  |
| --- | --- | --- | --- | --- | --- | --- | --- | --- | --- | --- |
|  |  |  |  | **Age(day)**  **(sep/Non-sep)** | **Gestational**  **age(sep/Non-sep)(weeks)**  **(mean ± SD)** | **Weight(sep/**  **Non-sep) (mean ± SD, g)** |  | **Septic group** | **Non-septic group** | |
| Wei et al,2015 [30] | China | Janu,2012-Oct,2014 | case  control study | 15.7±3.6  16.1±4.1 | NA | NA | NA | N=50  1. Confirm diagnosis*   1. Clinical diagnosis§   Blood cultured Staphylococcus aureus in 24 cases (48.00%)  Staphylococcus epidermidis in 9 cases (18.00%)  Staphylococcus haemolyticus in 4 cases (8.00%)  Klebsiella pneumoniae in 3 cases (6.00%)  Escherichia coli in 3 cases (6.00%)  Enterobacter cloacae in 3 cases (6.00%)  Pseudomonas aeruginosa in 2 cases (4.00%)  Staphylococcus saprophytic in 2 cases (4. 00 %) | locally infected newborns:n=50  38 cases of bacterial pneumonia 12 cases of enteritis. | |
| Guo et al,2014 [31] | China | Janu,2011-Janu,2014 | case  control study | 14.6±0.3 | 34±1 | 2150±150 | NA | N=50  1. Confirm diagnosis*   1. Clinical diagnosis§ | Non-septic group:n=50 | |
| Ma et al,2012 [32] | China | July,2011-Janu,2012 | case  control study | 16.5±5.6/  3-29 | NA | NA | NA | N=32   1. Confirm diagnosis* 2. Clinical diagnosis§ | local infection: n=41  Non-infection: n= 65 | |
| Feng et al,2010  （excluded） | China | Jan,2006-Aug,2009 | case  control study | NA | NA | NA | NA | N=37  1. Confirm diagnosis*  2. Clinical diagnosis§ | local infection: n=52  Healthy newborn:n=30 | |

*Clinical manifestations and meet any of the following: (1) blood culture or sterile body cavity culture of pathogens; (2) if the blood culture specimens cultivate pathogens, then must be with another (The same species of bacteria is cultured in blood, or in a sterile body lumen, or in a catheter tip).

§clinical manifestations and any of the following: (1)non-specific examination ≥ 2. (2) positive blood test antigen or DNA test

**Table 2 continued**

| **Author,**  **publication year** | **Country, Region** | **Study period** | **Study design** | **Study population** | | | **Sepsis onset** | **Characteristics and number of patients** | |  |
| --- | --- | --- | --- | --- | --- | --- | --- | --- | --- | --- |
|  |  |  |  | **Age(day)**  **(sep/Non-sep)** | **Gestational**  **age(sep/Non-sep)(weeks)**  **(mean ± SD)** | **Weight(sep/**  **Non-sep) (mean ± SD, g)** |  | **Septic group** | **Non-septic group** | |
| Chiara Poggi et al,2015 [35] | Europe | May 2013 to April 2014 | Cohort study | 25±19/28±19 | 25.6±2.0/28.8±2.0 | 684±215/1021± 233 | LOS:19 | Infants who developed possible LOS  (LOS group) | the next infant born who did  not develop signs and symptoms of  infection and fulfilled the inclusion  criteria was enrolled | |
| Montaldo et al,2017 [36] | Europe | January 2013 and  March 2016 | Case-control | NA | 29.9±1.1/30.2 ±1.4 | 1082±205/1102 ±192 | EOS | Proven sepsis was diagnosed in case of growth of a recognized pathogen in pure culture or-in case of a mixed growth or growth of a skin commensal-the acute onset of >3 predefined clinical signs was considered | The uninfected group included those neonates who did not show  any clinical, radiographic, or laboratory findings attributable to  sepsis, or showed findings fully explained by another diagnosis. | |
| Jehan H Sabry et al,2016 [37] | Africa | November 2015 to April 2016 | Case-control | NA | 36.1±2.36/38.02±1.31 | 2590±620/2840/830 | LOS | Sepsis was diagnosed according to Griffin Neonatal Sepsis Score in which Feeding intolerance | Inclusion criteria included maternal history of fever or resistant UTI, immune suppressant drugs intake, history of PROM or bad odor of amniotic fluid, regardless mode of delivery, number, or sex. | |
| Ozdemir et al,2016 [38] | Asia | October 2015 and February 2016 | Cohort study | NA | 39.12±0.93 (39)  /39±0.98 (39) | 3286.4±460.89/3379.33±420.93 | EOS | High probable sepsis: 1.At least three sepsis-related clinical signs; 2.CRP > 6 mg/L; 3.At least two other altered serum parameters in addition to CRP; 4.Blood culture: Positive or negative  Probable sepsis: 1.less than three sepsis-related clinical signs; 2.CRP > 6 mg/L; 3.At least two other altered serum parameters in addition to CRP; 4.Blood culture: negative  Possible sepsis: 1.less than three sepsis-related clinical signs; 2.CRP > 6 mg/L; 3.less than two other altered serum parameters in addition to CRP; 4.Blood culture: negative | No Sepsis: 1.No sepsis-related clinical signs; 2.CRP < 6 mg/L; 3.No altered serum parameters in addition to CRP; 4.Blood culture: negative | |
| Topcuoglu et al,2016 [39] | Asia | January and October 2014. | Cohort study | NA | 28.4±2.6/28.9±2.8 | 1084±318/1140±338 | LOS | Töllner’s sepsis score: Points are given for each parameter (0, 1, 2, or 3); a higher number of points reflects greater severity. According to this scoring system, a score of >10 indicates clinical sepsis, a score of <5 indicates no sepsis, and a score of 5–10 indicates possible sepsis. | newborns without clinical findings of sepsis served as controls. | |
| Neeraj Kumar et al,2018 [40] | Asia | March 2015  through October 2016 | cross-sectional study | NA | Case:  Preterm(<37 wk):22(53.6)  Term(≥37wk):19(46.3)  Control:  Preterm(<37wk):23(56)  Term(≥37wk):18(43.9) | Case:  <2.5kg:29(70.7)  ≥2.5kg:12(29.2)  Control:  <2.5kg:27(65.8)  ≥2.5kg:14(34.1) | LOS+EOS | Neonates with ≥1 features of sepsis  and/or two risk factors were included. | with no clinical and laboratory evidence of sepsis | |
