# Supplementary figures and images for: The combination of procalcitonin and C-reactive protein or presepsin alone improves the accuracy of diagnosis of neonatal sepsis: a meta-analysis and systematic review
Source: Crit Care. 2018 Nov 21;22:316. doi: 10.1186/s13054-018-2236-1 (PMC6249912; doi:10.1186/s13054-018-2236-1)

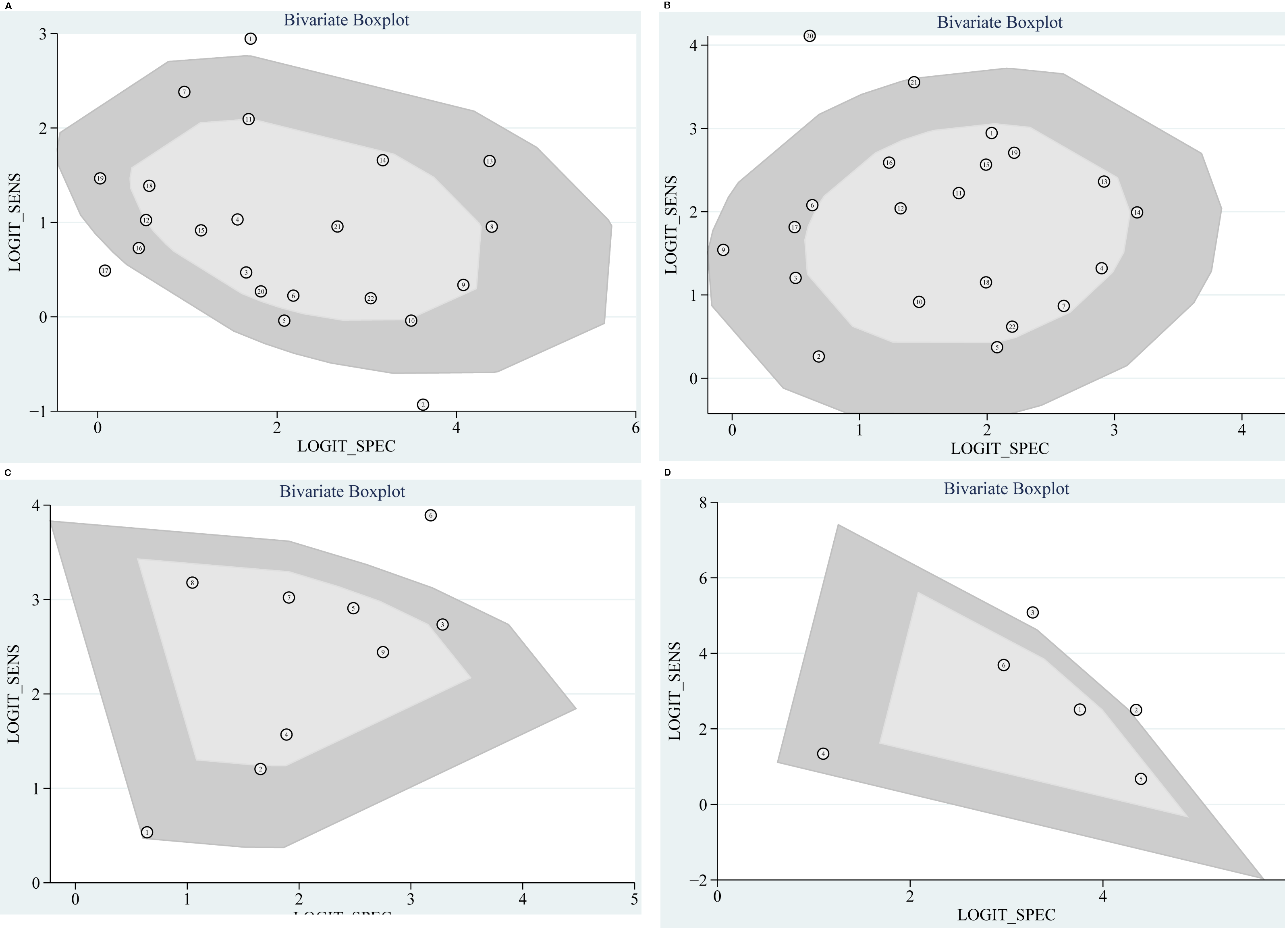

Supplement: Supplementary file 2 — Figure S1. Bivariate boxplots. Bivariate boxplots of CRP (A), PCT (B), PCT plus CRP (C), and presepsin (D). (TIF 2191 kb) [file 13054_2018_2236_MOESM2_ESM.tif]

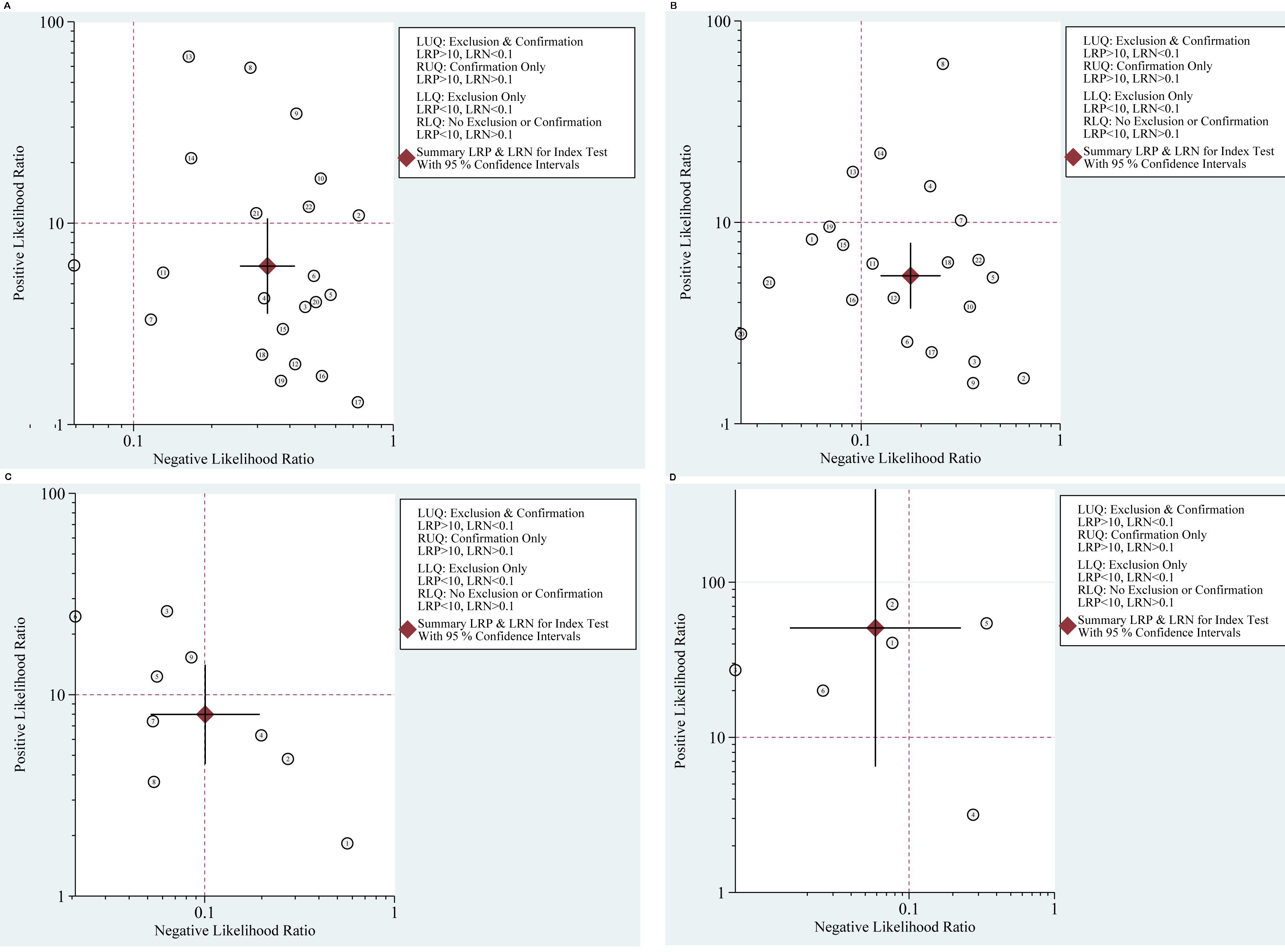

Supplement: Supplementary file 3 — Figure S2. Likelihood ratio scattergrams. Scattergrams evaluating the positive likelihood ratios in the diagnosis of neonatal sepsis for CRP (A), PCT (B), PCT plus CRP (C), and presepsin (D). (TIF 2591 kb) [file 13054_2018_2236_MOESM3_ESM.tif]

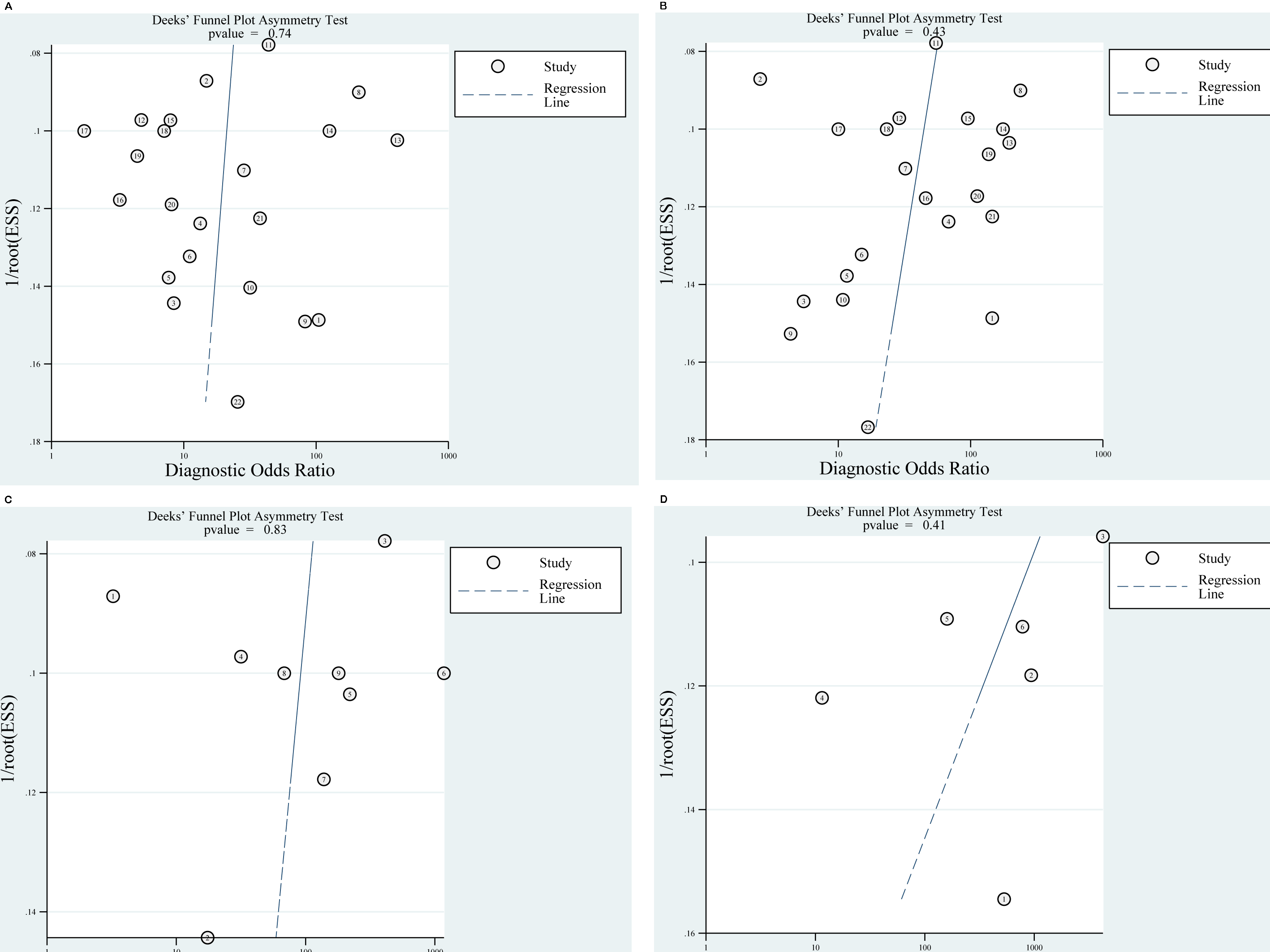

Supplement: Supplementary file 4 — Figure S3. Deek’s funnel plots. Funnel plots evaluating publication bias of CRP (A), PCT (B), PCT plus CRP (C), and presepsin (D). (TIF 1861 kb) [file 13054_2018_2236_MOESM4_ESM.tif]

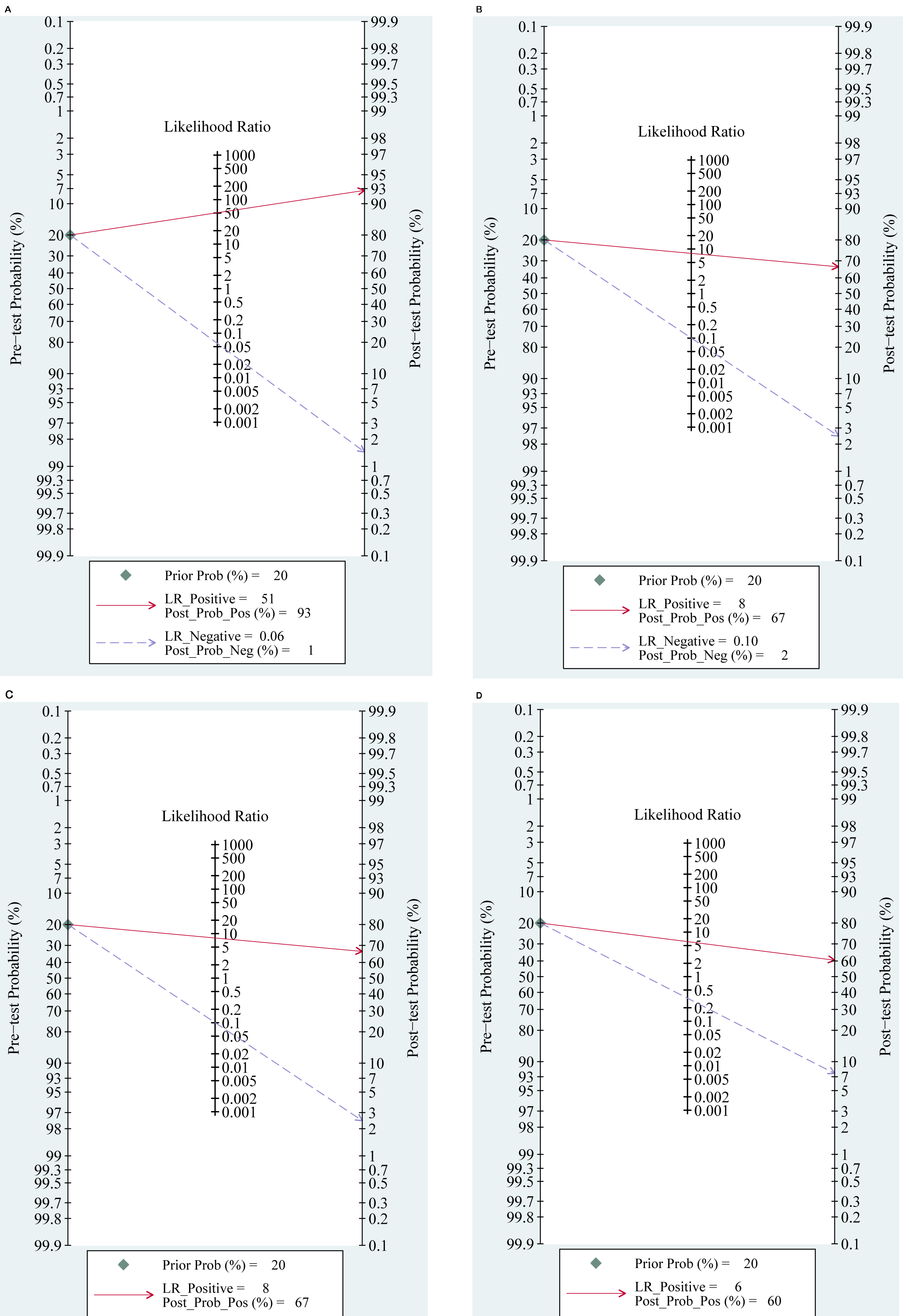

Supplement: Supplementary file 5 — Figure S4. Fagan diagram. A, Fagan diagram of CRP. B, Fagan diagram of PCT. C, Fagan diagram of PCT plus CRP. D, Fagan diagram of Presepsin. (TIF 3370 kb) [file 13054_2018_2236_MOESM5_ESM.tif]
